# Naturally Occurring TPE-CA Maintains Gut Microbiota and Bile Acids Homeostasis *via* FXR Signaling Modulation of the Liver–Gut Axis

**DOI:** 10.3389/fphar.2020.00012

**Published:** 2020-02-06

**Authors:** Linlin Liu, Zhenli Liu, Hui Li, Zhiwen Cao, Wen Li, Zhiqian Song, Xiang Li, Aiping Lu, Cheng Lu, Yuanyan Liu

**Affiliations:** ^1^School of Chinese Materia Medica, Beijing University of Chinese Medicine, Beijing, China; ^2^Institution of Basic Theory, China Academy of Chinese Medical Sciences, Beijing, China; ^3^Institute of Basic Research in Clinical Medicine, China Academy of Chinese Medical Sciences, Beijing, China; ^4^School of Chinese Medicine, Hong Kong Baptist University, Hong Kong, Hong Kong

**Keywords:** antibiotics, total phenolic extracts of *Citrus aurantium* L., dysbiosis, intestinal barrier integrity, bile acid, liver–gut axis

## Abstract

Antibiotics-induced changes in intestinal flora (dysbiosis) may have various effects on the host. Dysbiosis is associated with numerous metabolites including bile acids, which are produced in the liver from cholesterol and metabolized in the gut by intestinal microbiota. Total phenolic extracts of *Citrus aurantium* L. (TPE-CA) are rich in dietary flavanones and their glycosyl derivatives, including flavones, flavonols, polymethoxyflavones and coumarins, which exert positive health effects on the microbiota. The aim of this study is to elucidate the interplays between the intestinal microbiota and bile acids metabolism attributed to antibiotics. Mice were exposed to broad-spectrum antibiotics, such as ampicillin, streptomycin and clindamycin, for 14 days. This exposure resulted in reduced bacterial diversity and richness, and destroyed intestinal permeability. The homeostasis of bile acids was also affected. Subsequent TPE-CA administration, counteracted most of the dysbiosis, and reshaped intestinal permeability, these effects occurred *via* upregulation of zonula occludens 1 and occludin associated proteins and downregulation of serum endotoxin compared to the antibiotics group. TPE-CA maintained the homeostasis of bile acids *via* modulation of the liver–gut axis related farnesoid X receptor (FXR)/fibroblast growth factor 15 (FGF15) pathway and FXR-targeted protein. Our findings indicated that TPE-CA exerted a protective effect on the restoration of intestinal microbiota composition, reshaped barrier integrity and maintained bile acid homeostasis *via* the liver–gut axis with antibiotics-induced dysbiosis.

## Introduction

The gut microbiota is an indispensable “metabolic organ” that participates in nutrient processing and the production of essential compounds, such as short-chain fatty acids and bile acids, and it contributes to gastrointestinal system maturation and immune system shaping (Lin et al., 2018). Numerous endogenous and exogenous factors affect microbial composition, such as the host's physiology, immunity, diet, antibiotics and environmental factors. (Dethlefsen et al., 2006; Swann et al., 2011a).One of factors, namely antibiotics, is widely used for bacterial infections. However, mounting evidence demonstrates that antibiotics adversely impact the host physiology and alter the intestinal flora, which is known as “dysbiosis” (Jernberg et al., 2007; Ju et al., 2017).

Broad-spectrum antibiotics affect the overall abundance of microbial composition and cause a rapid decline in diversity, evenness, and taxonomic richness (Rea et al., 2011). Evidence showed that broad-spectrum antibiotics, such as ampicillin, streptomycin and vancomycin, promoted dysbiosis in models (Antonopoulos et al., 2009; Ubeda et al., 2010). The microbiota plays a role in the maintenance of intestinal barrier integrity. Intestinal barrier integrity prevents microbiota endotoxin product translocation from the intestinal to the liver. Tight junctions, such as zonula occludens 1 (ZO-1) and occludin control paracellular permeability and protect the integrity of the intestinal barrier. Antibiotics were used increased the incidence of gastrointestinal diseases, and interferes with the intestinal homeostasis and disrupts intestinal barrier integrity (Deng et al., 2018). Restoring the composition of microorganisms and strengthening of intestinal barrier integrity are essential to restore intestinal homeostasis. The metabolism of bile acids is consecutively disturbed due to intestinal bacteria alterations (Swann et al., 2011b). Emerging dietary strategies such as probiotics, prebiotics, and polyphenols recommended modulation of the composition of the human gut microbiota (Lee et al., 2006).

Citrus fruits contain a large number of polyphenols, which have a protective effect on human physiology (Zhao et al., 2018). Many citrus fruits are used as medicines such as *Citrus aurantium* L., and exert antioxidative, anti-inflammatory, and hepatoprotective effects. The total phenolic extracts of *C. aurantium* L. (TPE-CA) were prepared in our laboratory, and primarily contain dietary flavonoids and their glycosyl derivatives, flavones, flavonols, polymethoxyflavones and coumarins. Our recent research revealed that, TPE-CA exhibited hepatoprotective effects on exogenetic chemical induced hepatic injury *via* modulation of the cytochrome P450 enzymes (Shu et al., 2017; He et al., 2019). Bile acid synthesis occurs in the liver, *via* the cytochrome P450 enzymes (CYPs). Cholesterol 7α-hydroxylation (CYP7A1) and sterol 12α-hydroxylase (CYP8B1) are the rate-limiting enzyme for bile acids produced, which determines the content of bile acids and the ratio of CA and CDCA, respectively (Li and Chiang, 2014). The literature reveals early quercetin from *Citrus* treatment has a good intervention on changes of the gut microbiota and developing gastroenteritis (Jin et al., 2010; Minamisawa et al., 2017). Overall, TPE-CA as a naturally occurring extract, may produce benefits on human health. Bile acids are produced in the liver and further metabolized in the intestine by gut microbiota. Numerous studies evaluated the link between changes in bile acids composition and antibiotics induced dysbiosis (Swann et al., 2011b). Farnesoid X receptor (FXR) is highly expressed in liver and gut, and it regulates protein expression of bile salt export pump (BSEP), Na+-taurocholate cotransporting polypeptide (NTCP) and organic anion transporters (OATPs), which are transporters and enzymes that involved in regulating bile acid homeostasis (Kriaa et al., 2019). Activated FXR in the intestine induces endocrine hormone fibroblast growth factor 15 (FGF15), which decreases the transcription of CYP7A1 and CYP8B1to limit the synthesis of bile acids. A previous study demonstrated that auraptene from the peels of *Citrus* regulated liver metabolism *via* FXR (Gao et al., 2018). Therefore, FXR was identified as a novel molecular target for the treatment of bile acid disorders. CA and CDCA conjugates with amino acids (glycine or taurine) in hepatocyte are stored in the gallbladder and released into the intestine *via* the BSEP transporter (Chiang, 2009). Conjugated bile acids such as taurochenodeoxycholic acid (TCDCA), glycochenodeoxycholic acid (GCDCA), taurocholic acid (TCA) and glycocholic acid (GCA) are subjected to chemical modifications to produce secondary bile acids such as deoxycholic acid (DCA) and lithocholicacid (LCA) by bile salt hydrolase (BSH) from microbiota (Jones et al., 2008). Ninety five percent bile acids are re-absorbed by the sodium-dependent bile acid transporters (ASBT) at the end of the ileum, and reuptake bile acids into hepatocyte under NTCP and OATPs transporters. FXR and the G protein-coupled receptor 1 (TGR5) are bile acids receptors that regulate energy metabolic, glucose metabolic, lipid metabolic and anti-inflammatory and other host processes (Lefebvre et al., 2009).

Therefore, it is increasingly important to understand the factors that result in the alteration of gut microbiota and the potential treatment strategies to restore the gut ecology and bile acids metabolism. The liver-intestinal axis connects the liver with the intestine, and it may provide a new perspective for determining the treatment of liver and intestinal diseases. We investigated the liver–gut axis related FXR/FGF15 signaling and FXR modulated transporter proteins to investigate the effects of TPE-CA on antibiotics induced dysbiosis, especially the interplay between microbiota and bile acids.

## Materials and Methods

### Materials and Reagents

Ammonium formate and formic acid (HPLC grade, Lot. 095224) were obtained from MREDA Technology Inc. (USA). Methanol and acetonitrile (HPLC grade) were purchased from Fisher Scientific (Fair Lawn, NJ, USA). The deionized water was taken from Millipore Milli-Q Water Purification System (Millipore, USA). Standard substances of cholic acid (CA), chenodeoxycholic acid (CDCA), beta-muricholic acid (β-MCA), glycolithocholic acid (GLCA), ursodeoxycholic acid (UDCA), hyodeoxycholic acid (HDCA), deoxycholic acid (DCA), tauroursodeoxycholic acid (TUDCA), glycocholic acid (GCA), taurolithocholic acid (TLCA), glycochenodeoxycholic acid (GCDCA), taurocholic acid (TCA), lithocholic acid (LCA), glycohyodeoxycholic acid (GHDCA), and glycoursodeoxycholic acid (GUDCA), taurochenodeoxycholic acid (TCDCA), ampicillin, streptomycin and clindamycin were purchased from Shanghai Source Leaf Biological Technology Co., Ltd. (Shanghai, China). The purity of the above reagents was higher than 98%. Probiotic was obtained from Guangzhou Baiyunshan Pharmaceutical Holdings Company Limited (Guangzhou, China) Anti-CYP7A1 (ab65596), anti-CYP8B1 (ab191910), anti-OATPs (ab224610), anti-SLC10A1 (NTCP, ab131084), anti-ABCB11 (BSEP, ab155421), anti-NR1H4 (FXR, ab235094), anti-FGf15 (ab229630), anti-Zonula occludens 1 (ab96587), anti-occludin (ab167161) and anti-GAPDH (ab8245) antibodies were obtained from Abcam (USA). BCA protein assay kit was purchased from Beyotime Institute of Biotechnology. Endotoxin assay kits were purchased from Xiamen Bioendo Technology Co., Ltd (Xiamen, China).

### Preparation of TPE-CA

The fruit of *C. aurantium* L. was purchased from Jiangxi province. The detailed extract procedure of *C. aurantium* L., including the extraction solvent, extraction method and time were optimized in our previous study (Li et al., 2018). The present study soaked 400 g of three smashed power with 1 l ethanol for 30 min. The mixture was boiled for 1 h three times. The aqueous extracts were mixed and concentrated in a vacuum rotary evaporator and dried in a vacuum oven, 132 g of dry phenolic extract was obtained. The prepared total phenolic extracts of *C. aurantium L*. (TPE-CA) including 32 polyphenol compounds, there were quantified and validated using HPLC-DAD/UV and RRLC-QqQ-MS instruments in the previous studies (Shu et al., 2017; Zhao et al., 2018). The specific contents of 32 phenolic compounds are recorded in the **Table S1**, **Figure S1**. The TPE-CA samples were stored at −20°C in a freezer and brought to room temperature before use (He et al., 2019).

### Animal Experiments

#### Animals

Forty healthy male C57BL6 mice (weighing 20 ± 2 g) were purchased from Beijing Vital River Laboratory Animal Technology Co., Ltd. All animals were housed in a specific pathogen-free (SPF) grade environment. Mice were *ad libitum* fed with lab chow and water under temperature-controlled room (22 to 25°C) with automatic control of 12-hour alternating light/dark cycle (6.30 am–6.30 pm). All animals' procedures were approved by the Experimental Animal Ethics Committee of Basic Theory, China Academy of Chinese Medical Sciences.

#### Antibiotics-Induced Mice Model

Ampicillin, streptomycin and clindamycin were mixed into sterile drinking water a final concentration of 1 mg/ml as described previously (Lamouse-Smith et al., 2011). The drinking solution containing the antibiotics was available for two weeks *ad libitum*. Replaced and/or refreshed water every 3 days for the duration of the experiment. Antibiotics-induced dysbiosis was evaluated *via* alterations in the microbiota, gut permeability and endotoxin. The weight of each mouse was recorded weekly.

#### Experimental Design

Mice were acclimated for one week in quarantine then randomly divided into four groups of 10 animals each, normal control group, antibiotics-induced model group, TPE-CA treated group, and probiotic positive control group. The normal control given normal food and water, and model group received mixture antibiotics at a concentration of 1 mg/ml for two weeks. The TPE-CA treated group received 8.7 g/kg TPE-CA extract (26 g/kg crude drug) *via* oral gavage once daily for four weeks. The dose of TPE-CA was based on previous experimental work in our laboratory (Shu et al., 2017). The positive treated group received probiotics (*Lactobacillus casei* DG, 10^9^ cells in water, 100 µl) for four weeks (Guida et al., 2018; Shi et al., 2018b). The weight of each mouse was recorded weekly throughout the experimentation, and the water was replaced every 3 days for the duration of the experiments.

### Samples Collection

Feces pellets were collected before sacrificed. Liver, cecal content and ileum samples were collected on sacrifice, weighted and immediately frozen in liquid nitrogen, and stored separately at −80 °C. Blood samples were also collected and transferred into vacuum blood collection tube. In addition, after the sacrifice, the weight of each mice and its cecal tissues were measured.

### Microbial Community Analysis

#### DNA Extraction

Cecal contents (100 mg) were collected and immediately frozen at −80 °C upon extraction. The total intestinal bacterial DNA was isolated using a PowerSoil DNA Isolation Kit (MoBio Laboratories, Carlsbad, CA) following the manufacturer's instructions. The purity and quality of the genomic DNA were checked by using a NanoDrop 2000 Spectrophotometer (Thermo, USA) and 0.8% agarose gels.

#### PCR Amplification of 16S rRNA V3–V4 Region

Polymerase chain reaction (PCR) amplified the bacterial 16S rRNA gene V3–V4 hypervariable region the primers 338F (ACTCCTACGGGAGGCAGCAG) and 806R (GGACTACHVGGGTWTCTAAT) (Munyaka et al., 2015). For each cecal content sample, 10-digit barcode sequence was added to the 5' end of the forward and reverse primers (provided by Allwegene Company, Beijing) to distinguish PCR products. PCR was perform on Mastercycler Gradient using 25 µl reaction volumes containing 12.5 µl 2× Tag PCR Master Mix, 3 µl BSA (2 ng/µl), 2 µL Primer (5 µM), 2 µl template DNA, and 5.5 µl ddH_2_O. Cycling parameters were 95 °C for 5 min, followed by 32 cycles of 95°C for 45 s, 55°C for 50 s and 72°C for 45 s with a final extension at 72°C for 10 min. Three PCR products were polymerized for each sample to mitigate reaction-level PCR biases. The PCR products were purified using a QIA quick Gel Extraction Kit (QIAGEN, Germany), quantified using Real Time PCR, and sequenced at Allwegene Company, Beijing.

#### High-Throughput Sequencing.

Deep sequencing was performed on Miseq platform at Allwegene Company (Beijing). After the run, image analysis, base calling and error estimation were performed using Illumina Analysis Pipeline Version 2.6.

### RRLC-QqQ-MS ^n^ Analysis of Bile Acids Composition in Liver and Feces

Liquid chromatographic separation and mass spectrometric detection were performed RRLC-QqQ-MS ^n^ system from Agilent, including a G1311A binary pump, a G1311A vacuum degasser and a G1311A autosampler and triple quadrupole mass spectrometer equipped with an electrospray source (Series 6410, Agilent Technologies). The chromatographic separation was performed on a Poroshell SB-C18 column (4.6 mm × 100 mm; 2.7 µm). The mobile phase consisted of 10 mM ammonium formate aqueous solution (0.1% v/v formic acid) (A) and acetonitrile (B). The gradient elution program (A/B, v/v) was performed as follows: 0 min, 40:60%; 7.6 min, 15:85%; 14.5 min, 0:100%; from 14.5 to 25min, the mobile phase B was maintained at 100%. The injection volume of all samples was 2 μl. The column temperature was set at 25°C and flow rate was 0.3 ml/min.

The mass spectrometer was programmed in the negative ion mode to produce MS/MS spectra by ESI source. The parameters were as follows: spray voltage, 4.0 kV; capillary temperature, 400°C; drying gas flow rate, 11 l/min; corona current of 10 nA, and nebulizer pressure, 45 psi; sheath gas temperature, 250°C and sheath gas flow rate, 7 l/min. Scan width for MRM, the MRM ion pairtransitions and collision energy levels of each component are listed in online resources **Table S2**.

### Preparation of Samples

Liver samples were extracted by protein precipitation method. Approximately 200 mg of liver was thoroughly ground in liquid nitrogen and transferred into 10 ml tube, 6 ml of acetonitrile was added. The tube was agitated for 20 s using a vortex mixer. Then, the tubes were treated with ultrasonic processing for 30 min. After cooling in ice bath for 10 min, the samples were centrifuged at 10,000×*g* for 10 min at 4°C. The supernatants were collected into a clean tube. This extraction procedure was repeated twice. The extract was blow-dried under nitrogen and redissolved with 100 ul of 50% acetonitrile and was then centrifuged at 10,000×*g* and 4°C for 10 min. After filtering through 0.22 µm-micropore membranes, the samples were stored at 4°C prior to use.

Feces samples were freeze dried in a vacuum freeze dryer and pulverized to obtain powders. Approximately 200 mg of feces were weighted into a 10 ml tube, 2 ml of acetonitrile was added. The mixture was done in the same manner as the following preparation of liver samples.

### Preparation of Stocks, Calibration Standards Samples, and Quality Control Samples

Each of the standard substances of bile acids were accurately weighed and dissolved individually in methanol, the stock solutions concentrations of 0.99, 1.01, 1.00, 0.98, 1.01, 1.04, 0.97, 0.99, 1.02, 1.00, 1.01, 0.49, 0.45, 0.99, 1.01 and 1.00 mg/ml for CA, LCA, DCA, CDCA, β-MCA TCDCA, GCDCA, TCA, GCA, HDCA, GHDCA, TLCA, UDCA, TUDCA, GUDCA and GLCA, respectively. Prior to LC–MS analysis, calibration standard solutions were prepared by spiking 10 µl of working standard solutions with 190 µl blank liver/feces homogenate. Quality control sample was prepared by pooling a same volume from each calibration standard liver/feces sample. The individual standard solutions were stored at −20°C freezer.

### Method Validation

#### Selectivity

The selectivity was evaluated *via* comparison of the chromatograms of a blank liver/feces sample, a blank liver/feces sample spiked with the working solutions, and the liver/feces samples in the RRLC-QqQ-MS ^n^ system.

#### Linearity and Lower Limit of Quantification (LLOQ)

The calibration curves for each compound were constructed by least-squares linear regression of the peak area versus the concentrations. Standard compound calibration curves consisted of six concentration levels, which are analyzed in triplicate according to the established method. Standard curves were in the form of Y = aX + b. The linearity was considered acceptable if the squared correlation coefficient (R^2^) was >0.99 for each calibration curve. LLOQ was defined as the minimum concentration that can be reliably quantified based on the signal-to-noise ratio of 10:1 and reproduced with precision and accuracy less than 20% and 80–120%, respectively.

#### Precision and Accuracy

Six quality control samples were replicate analyzed at three levels of concentration. The precision and accuracy of the method were validated by the determination of the intra- and inter-day variances on the same day and for three consecutive days. To determine repeatability of precision and accuracy, the relative errors (RE) (%) within the nominal value of 85–115% and relative standard deviation (RSD) (%) within ±15% were taken to be the measure of reproducibility.

#### Recovery and Matrix Effect

The extraction recovery was determined by comparing the peak area of pre-spiked extracted liver/feces standard QC samples to those of post-spiked standards at equivalent concentrations, and three repetitions of QC samples were evaluated. To evaluate the matrix effects quantitatively, six replicates of each concentration of quality control samples were prepared. Then, according to the method of sample preparation and extraction, all samples were evaporated and dissolved in mobile phase The matrix effect was determined by comparing the peak response of the analytes in liver/feces samples with those of the pure standards prepared in the mobile phase.

#### Stability

The stability of the analyte in stored liver/feces was studied by the established method at different time points (1 month at −80°C, room temperature for 24 h, three successive cycles of freezing at −80°C). All experiments results were compared with results from freshly prepared and measured samples.

### Enzyme-Linked Immunosorbent Assay (ELISA)

Serum samples were collected before sacrifice and the concentrations of endotoxin (ET) were determined using ELISA kits (Xiamen Bioendo Technology Co., Ltd., Xiamen, China) in accordance with the manufacturer's instructions. The absorbance of each well was measured at 450 nm (test wavelength) using an ELISA reader (Bio-Tek Instruments, Winooski, VT, USA). The sample concentration was calculated by the standard curve and expressed in EU/ml. All experiments were repeated three times.

### Western Blot Analysis

Total proteins were extracted from liver and colon tissues using a protein lysis buffer (Beyotime, Shanghai, China) supplemented with a protease inhibitor cocktail (Amresco, Houston, TX, USA), and incubated on ice for 30 min followed by centrifugation, respectively. The protein content in the supernatant was determined by BCA protein assay kit (Beyotime, Shanghai, China) according to the manufacturer's instructions after centrifugation at 12,000×*g* for 15 min at 4 °C. Sodium dodecyl sulfate-polyacrylamide gel electrophoresis (SDS-PAGE) was separated by equal amounts of protein (20 µg) and transferred to polyethylene-difluoride (PVDF) membrane (Immobilon TM-P; Millipore, Massachusetts, USA). Membranes were blocked by 5% skimmed milk in TBST for 2 h at room temperature. Subsequently, membranes were probed with monoclonal primary antibodies: GAPDH (1:5,000 dilution), FXR (1:1,000 dilution), Occludin (1:2,000 dilution), ZO-1 (1:1,000 dilution), FGF15 (1:1,000 dilution), NTCP (1:2,000 dilution), OATPs (1:3,000 dilution), BSEP (1:2,000 dilution), CYP7A1 (1:1,000 dilution), and CYP8B1 (1:1,000 dilution) overnight at 4 °C. After three times of washing with TBST, membranes were incubated with the horseradish peroxidase-conjugated secondary antibodies (1:5,000) at room temperature for 2 h. Following washing, the protein bands were visualized by enhanced chemiluminescence detection reagents (Applygen Technologies Inc., Beijing, China). All the protein strips have been uploaded to the **Data Sheet 1**.

### BSH Enzyme Activity

The specific activity of BSH was determined *via* measurement of the release amount of amino acids in conjugated bile salts. TDCA was used as the substrate for BSH. Cecal contents were dissolved in an aqueous solution containing 20% glycerol and 1.8% sodium chloride at a 1:3 ratio (g: ml), diluted 10-fold in a peptone-yeast extract (PY) medium prepared as previously described (Zhao et al., 2014) and cultured at 37°C and 200×*g* for 12 h to allow anaerobic bacteria to proliferate. Then, 10 µl of PY medium (containing 50 µg/ml of TDCA) and 90 µl of the fermented cecal content were mixed in 1.5 ml eppendorf tubes, and incubated at 37°C for 30 min. The reaction was terminated with trifluoroacetic acid (15%, w/v). The mixture was centrifuged at 14,000×*g* for 2 min to remove the precipitate. The supernatants were transferred into a vial for RRLC-QqQ-MS ^n^ analysis of TDCA and DCA.

### Statistical Analysis

All data were obtained from three independent experiments and expressed as means ± standard deviation (SD). One-way analysis of variance (ANOVA) was used to compare the significant differences among, and Tukey's multiple comparison tests by using the GraphPad Prism 6.0 (GraphPad Software, Inc., San Diego, CA). LEfSe analysis of relative abundance of gut microbiota was based on Kruskal–Wallis and Wilcoxon tests, and the threshold on linear discriminant analysis (LDA) score was 4.0 to 5.0. Others were displayed by regular R packages. All analysis was performed by Graphpad Prism7.0. A threshold of *P <* 0.05 was considered as statistically significant.

## Results

### Effect of the Body Weight of Antibiotics-Induced Mice Following TPE-CA Treatment

**Figure 1:**
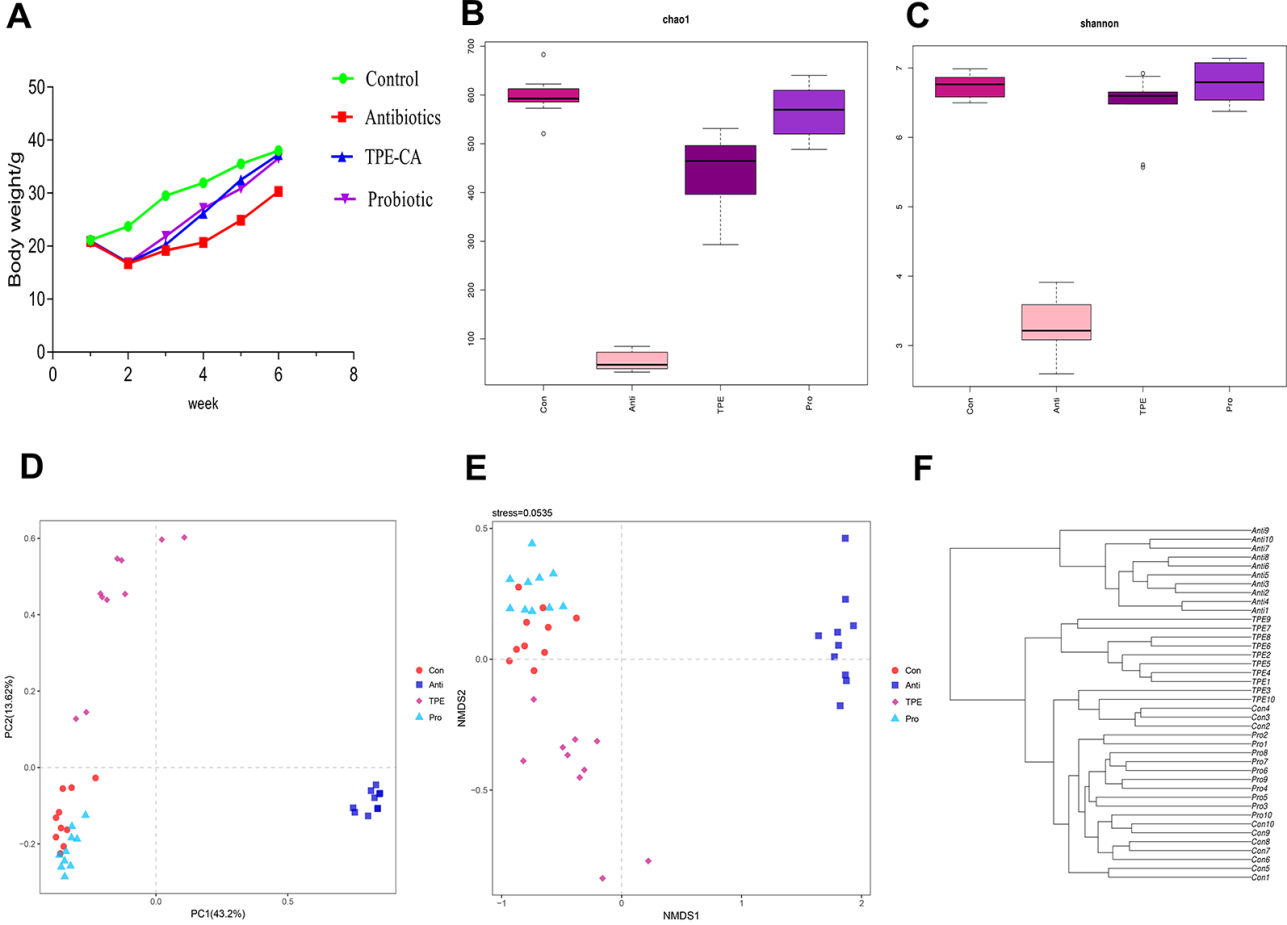
The effect of TPE-CA following antibiotics-induced dysbiosis *via* microbial diversity. **(A)** The body weight of mice for control group, antibiotics group, TPE-CA group and probiotic group. **(B)** Microbial α-diversity of cecal content samples indicated by chao1 index. **(C)** Microbial α-diversity of cecal content samples indicated by shannon Index. **(D)** Visualization of PCoA of unweighted UniFrac distances to show differences in bacterial community structure between samples from Control, Antibiotics, TPE-CA and Probiotic groups. Each point represents in a single sample. **(E)** Non-metric multidimensional scaling (NMDS) analysis with Bray–Curtis dissimilarity show differences in bacterial community structure between four groups. The distance between the points represents the level of difference; the closer the samples are, the higher the similarity between them. **(F)** Hierarchical clustering based on the Bray–Curtis similarity of cecal content microbial composition in Control, Antibiotics, TPE-CA and Probiotic groups. Ten animals per group.

The body weight was observed from 1 to 6 weeks. The mice suffered from weight loss and diarrhea and the weight lowest at week 2. Compared with the control group, the antibiotic group showed significant weight loss after treatment. TPE-CA group showed a significant increase in body weight compared to the antibiotics group. No difference between the TPE-CA group and the probiotics group (**Figure 1A**).

### Restoration of Intestinal Microbiota Disruption in Antibiotics-Induced Mice Following TPE-CA Treatment

#### Overall Composition Modulation of Gut Microbiota

We sequenced 16S rRNA genes from cecal contents after TPE-CA treatment in antibiotics induced dysbiosis to analyze the microbial composition and identify the effects of TPE-CA on intestinal microbiota structure. The specific data of 16s rRNA sequencing are shown in **Tables S6**, **S7**. Intestinal contents were examined at the level of phylum, class, order, family and genus(**Figures S2**–**S6**). The specific of genus_phylogeny for each groups are shown in **Figure S7**. The chao1 index and Shannon index with alpha-diversity were analyzed, and the results showed that microbial diversity in the antibiotics group were lower than in the control, indicating that the microbial diversity was decreased after antibiotics intervention (*P <* 0.01) (**Figures 1B, C**) and the specific data are shown in **Table S9**. Administration of TPE-CA or probiotic enhanced the level of alpha-diversity to the level of the antibiotics group (*P <* 0.01). Unweighted principle coordinate analysis (PCOA) was perform to visualize differences in bacterial taxa composition (**Figure 1D**), the results showed an significant separation between the four groups along the PC1 axis (43.2% of overall variation) with statistical significance, and the antibiotics group had a considerably altered microbial composition compared to the control group. The result of nonmetric multidimensional scaling (NMDS) analysis also displayed relative distinct clustering for each group (**Figure 1E**). The cluster of the TPE-CA group was closer to the probiotic group than the antibiotics group, indicating that TPE-CA treatment for four weeks could effectively restore the microbial community to the normal level after the destruction of antibiotics (**Figure 1F**).

#### Composition of Gut Microbial Community

**Figure 2:**
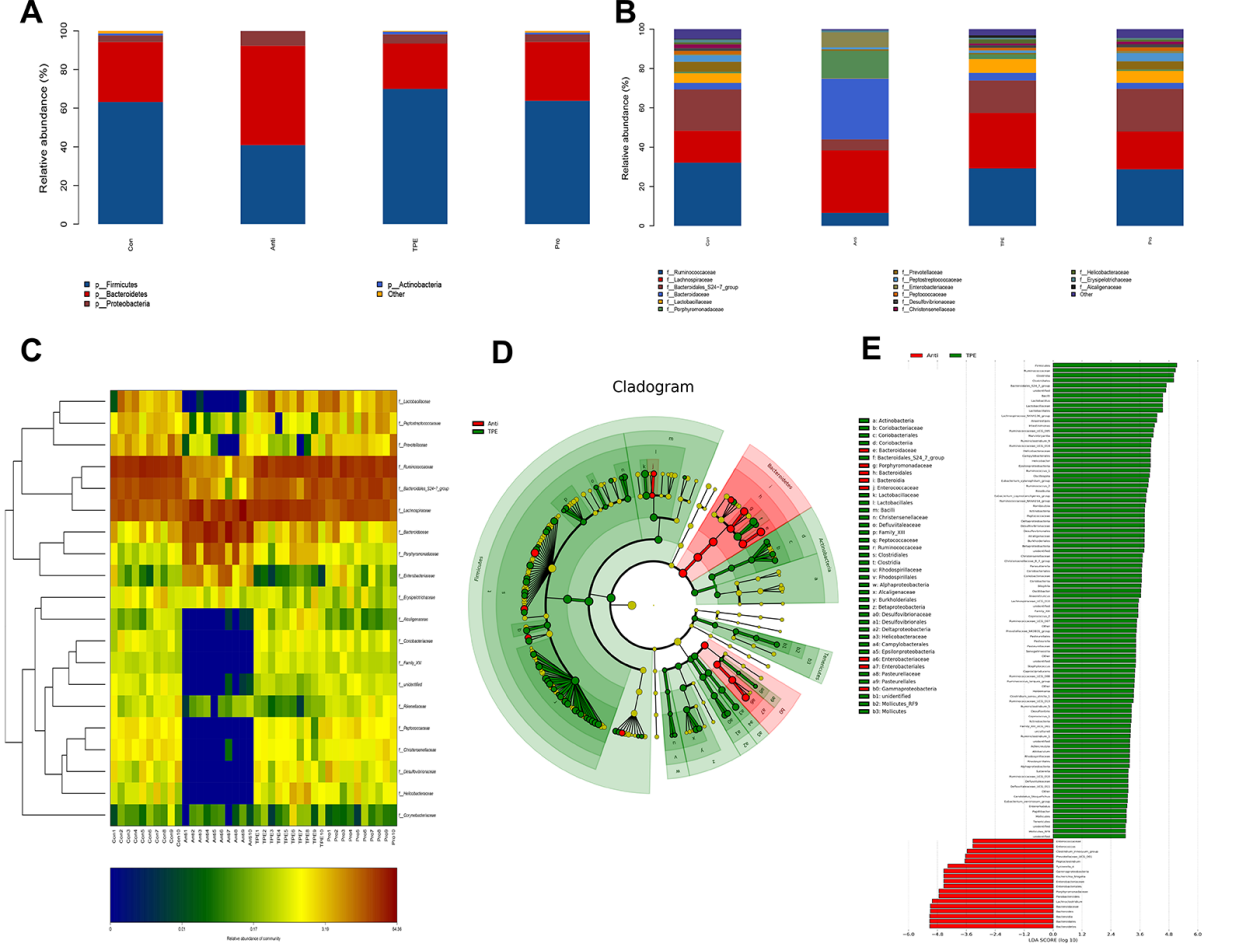
Effect of TPE-CA treatment on the composition of gut microbial community in antibiotics induced dysbiosis. **(A)** Relative abundance of bacterial phylum in Control, Antibiotics, TPE-CA and Probiotic groups. **(B)** Relative abundance of bacterial family in Control, Antibiotics, TPE-CA and Probiotic groups. **(C)** Heat map of differential bacterial taxa between four experimental groups. A range of colors, from blue to red indicates the prevalence of each bacterial. The left tree structure indicated the clustering information of each family. **(D)** Identification of significant differences in bacterial taxa between two experimental groups. Cladogram depicting differences and phylogenic location are shown. In each section, the diameter of the circle is proportional to the abundance of the taxon, green = taxon significantly enriched in antibiotics group, blue = taxon significantly enriched in TPE-CA group and yellow = non-significant. **(E)** LDA scores represent the degree of consistent difference in relative abundance between features in two groups of analyzed microbial communities. The threshold on (LDA) score was 4.0 to 5.0. The clades of the histogram identify statistical and biological differences between the communities. Green, indicating the antibiotics group; blue, indicating the TPE-CA group. Ten animals per group.

The abundance of the intestinal microbiota is presented as total operational taxonomic units (OTUs), the specific data are shown in **Table S8**, **Figure S8**. (Tao et al., 2019). At the phylum level, nearly 97% of the total bacteria belonged to 10 phylum, and the rest were allocated to other unclassified bacteria. The results showed dramatically increased *Bacteroidetes* and *Proteobacteria* and a decreased in *Fimicutes* and *Actinobacteria* in the antibiotics group compared to the control group (**Figure 2A**). However, the TPE-CA and probiotic groups were absolutely conversed against antibiotics group. At the family level, TPE-CA treatment significantly upregulated the decreased levels of *Ruminococcaceae*, *Bacteroidales_S24-7_group*, *Lactobacillaceae*, and *Prevotellaceae*, and remarkably suppressed the growth of *Lachnospiraceae*, *Bacteroidaceae*, *Porphyromonadaceae* and *Enterobacteriaceae* compared to the antibiotics group (**Figure 2B**).

To determine which genus was responsible for the differences in microbial composition between the four experimental groups, a characteristics comparison of the genus level was displayed on a heat map to represent the relative abundance of each family. Ordination of Bray–Curtis distances by taxonomic heat map (**Figure 2C**) and combined with the average clustering of statistically significant OTUs in control, antibiotics, TPE-CA and probiotic groups. The results revealed that *Bacteroides*, *Lachnoclostridium*, *Parabacteroides*, *Escherichia–Shigella*, *Tyzzerella_4* and *Subdoligranulum* were significantly associated with antibiotic use. The TPE-CA and probiotic groups had a significantly higher abundance of *Lactobacillus*, *Roseburia*, *Romboutsia*, *Ruminiclostridium_9*, *Lachnospiraceae_NK4A136_group* and *Ruminococcaceae_NK4A214_group* which were almost completely lost in the antibiotics group. The linear discriminant analysis effect size (LEfSe) method was used to elucidate the changes of major flora after TPE-CA treatment. The result showed that particular taxa were altered after antibiotics treatment, including *Bacteroidaceae*, *Porphyromonadaceae*, *Lachnoclostridium*, *Bateroidia*, *Enterobacteriaceae*, *Enterococcaceae and Gammproteobacteria*. In contrast, the intestinal microbiota including *Firmicutes*, *Clostridiales*, *Lactobacillaceae*, *Lactobacillus* and *Ruminococcus* were increased in TPE-CA group (**Figures 2D, E**).

### Improvement of Intestinal Integrity in Antibiotics-Induced Mice Following TPE-CA Treatment

**Figure 3:**
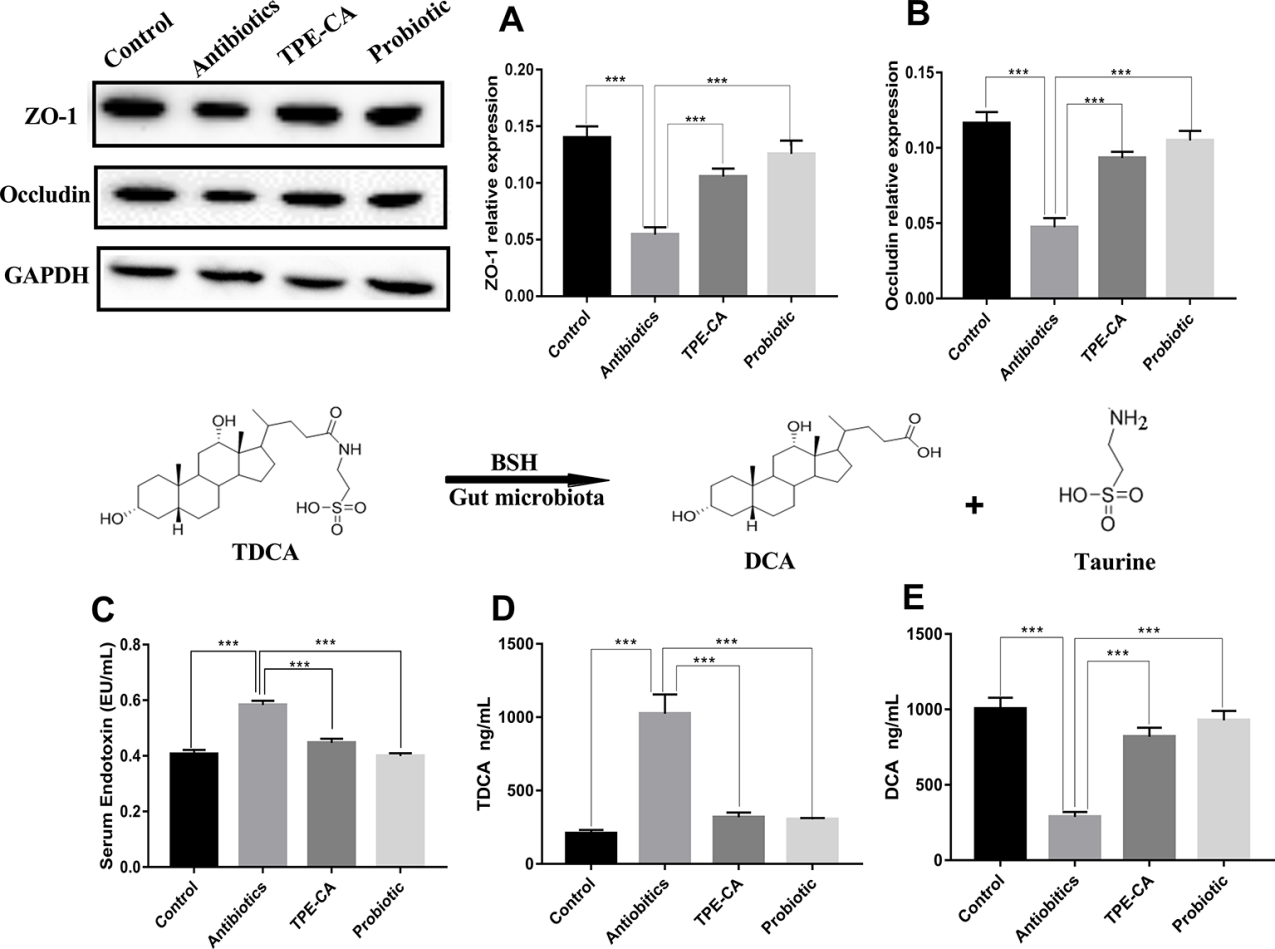
Improvement of TPE-CA treatment on intestinal integrity and on BSH activity of antibiotics- induced mice.TPE-CA group and probiotic group. **(A)** The level of ZO-1 for four groups. **(B)** The level of occludin for four groups. **(C)** The level of endotoxin in serumr for control group, antibiotics group, **(D)** The level of TDCA for control group, antibiotics group, TPE-CA group and probiotic group. **(E)** The level of DCA for control group, antibiotics group, TPE-CA group and probiotic group. ***p < 0.001. Values are represented as means ± S.D for 10 animals per group.

To further investigate the underlying intestinal permeability we measured the contents of ZO-1, occludin and endotoxin involved in dysbiosis (Xue et al., 2018). To further investigate the underlying intestinal permeability we measured the contents of ZO-1, occludin and endotoxin involved in dysbiosis. The enzyme activity of ZO-1 and occludin were decreased in the antibiotics group compared to the Control group (*p <* 0.01, **Figures 3A, B**), which demonstrates damage to mucosal integrity after antibiotics exposure. The antibiotics group showed significantly increased levels of endotoxin in serum, which indicated an increase in gut permeability after antibiotics treatment compared to the control group (*p <* 0.01, **Figure 3C**). After four weeks of treatment, the TPE-CA and probiotic groups exhibited significantly increased contents of ZO-1 and occludin, and drastically reduced contents of endotoxin. All the above results indicate that TPE-CA had stronger treatment effects on the intestinal permeability in mice.

### Effect of TPE-CA Treatment on BSH Activity

BSH functions for the deconjugation of conjugated bile acids (Jones et al., 2008). To determine BSH activity, we measured the levels of TDCA and DCA. As shown in **Figures 3D, E**. the level of DCA decreased and the level of TDCA increased in the antibiotics group compared to the control group. As expected, the levels of DCA and TDCA were conversely altered in the groups treated with TPE-CA or probiotic. This result indicated that TPE-CA increased the antibiotics- induces activity of BSH.

### Method Validation of Bile Acid Determination

No endogenous or extraneous peaks of interferences analytes were observed during the retention time, indicating the specificity of the method was acceptable. All of the calibration curves were linear within the test range, and the regression coefficient (R^2^) was >0.99. The lowest concentrations with RSD <20% were taken as LLOQs and were 24.31 ng/ml for CA, 16.34 ng/ml for LCA, 34.56 ng/ml for DCA, 83.86 ng/ml for CDCA, 45.32 ng/ml for β-MCA, 67.46 ng/ml for TCDCA,20.87 ng/ml for GCDCA, 10.52 ng/ml for TCA, 22.09 ng/ml for GCA, 5.69 ng/ml for HDCA, 13.24 ng/ml for GHDCA, 37.19 ng/ml for TLCA, 55.61 ng/ml for UDCA, 5.34 ng/ml for TUDCA, 29.47 ng/ml for GUDCA and 49.37 ng/ml for GLCA, respectively, which can be seen in **Table S3**. The accuracy and precision were analyted, the results showed that the accuracy was 87.40–104.72% (RE), the inter- and intra-day precision were 3.97–13.45% and 3.24–12.57% (RSD). The average recoveries of the analytes was 86.89–103.30% (RSD <15%), and the matrix effect was 87.94–103.59% (RSD <15%). These results showed that acetonitrile is a feasible and suitable medium for extracting analytes, and that no obvious matrix effect. In addition, the analytes of stored at room temperature (20 °C), −80 °C for 1 month, three freeze–thaw cycles were all stable with an accuracy of 99.32–104.37%. All consequences are shown in **Table 1**. In summary, a rapid, sensitive, accurate and specific method for the quantitative-profiling of 16 bile acids in mice liver and feces was developed and validated.

**Table 1:** The accuracy (intra- and inter-day), precision (intra- and inter-day), recovery, matrix effects, and stability for 16 compounds in mice (n = 6).

Compounds	Accuracy (%) n = 6		Precision (RSD, %) n = 6		Recovery		Matrix Effect		Auto-sampler for 24 h		At −80 ℃ for 1 Month		Freeze–thaw Cycles	
	Intra-day	Inter-day	Intra-day	Inter-day	Accuracy (%)	Precision (%)	Accuracy (%)	Precision (%)	Mean (%)	RSD (%)	Mean (%)	RSD (%)	Mean (%)	RSD (%)
tauroursodeoxycholic acid	95.36	98.09	7.56	6.27	89.19	8.83	99.09	6.29	99.32	5.44	99.51	3.03	100.74	5.48
taurochenodeoxycholic acid	90.42	92.78	12.76	4.34	97.78	3.18	103.23	3.41	101.22	10.67	103.19	3.52	99.57	6.97
taurocholic acid	97.63	98.99	7.90	5.97	92.23	3.13	102.50	5.31	100.82	4.95	100.15	9.39	98.27	5.45
taurolithocholic acid	102.82	91.61	11.59	4.63	99.97	6.02	88.79	6.54	103.31	10.97	99.89	9.92	99.52	5.62
cholic acid	95.84	101.32	7.50	7.32	92.23	9.84	101.34	10.65	98.83	3.28	97.98	10.47	97.59	6.75
chenodeoxycholic acid	87.40	89.53	4.84	9.81	103.30	7.78	93.22	7.74	95.82	9.19	96.16	3.92	97.92	6.84
ursodeoxycholic acid	90.07	98.47	11.99	4.48	99.97	8.10	90.18	10.16	96.85	9.22	98.24	8.31	98.09	5.44
hyodeoxycholic acid	104.10	94.66	6.06	12.57	86.89	5.07	100.11	7.20	100.43	4.92	99.92	3.95	98.48	4.79
deoxycholic acid	89.52	96.09	7.25	10.92	96.83	5.80	95.69	7.34	101.62	3.85	97.95	8.34	96.93	10.83
beta-muricholic acid	98.94	92.92	8.48	6.12	100.07	3.09	99.37	6.78	95.95	8.19	99.53	4.93	100.98	3.14
lithocholic acid	91.70	104.72	13.48	3.24	94.44	8.56	100.24	11.02	101.28	9.39	97.83	4.26	103.25	6.24
glycochenodeoxycholic acid	93.37	90.07	5.16	6.04	98.31	4.90	87.94	4.32	104.33	5.31	97.80	3.18	101.10	6.73
glycoursodeoxycholic acid	98.37	95.36	10.27	10.73	97.50	7.86	90.37	9.11	98.61	3.50	98.84	10.16	96.30	11.6
glycocholic acid	89.42	97.84	6.05	10.87	99.68	5.52	103.59	4.21	95.93	7.04	104.37	8.52	98.85	5.66
glycohyodeoxycholic acid	102.15	98.75	3.97	3.97	95.61	4.89	96.66	3.42	98.68	3.80	97.86	5.14	101.24	10.98
glycolithocholic acid	92.99	96.89	9.24	7.96	95.96	7.25	88.77	5.12	101.37	7.89	96.36	5.50	99.33	10.46

### Restoration of Antibiotics Disruption of Enterohepatic Circulation of Bile Acids With TPE-CA Treatment

**Figure 4:**
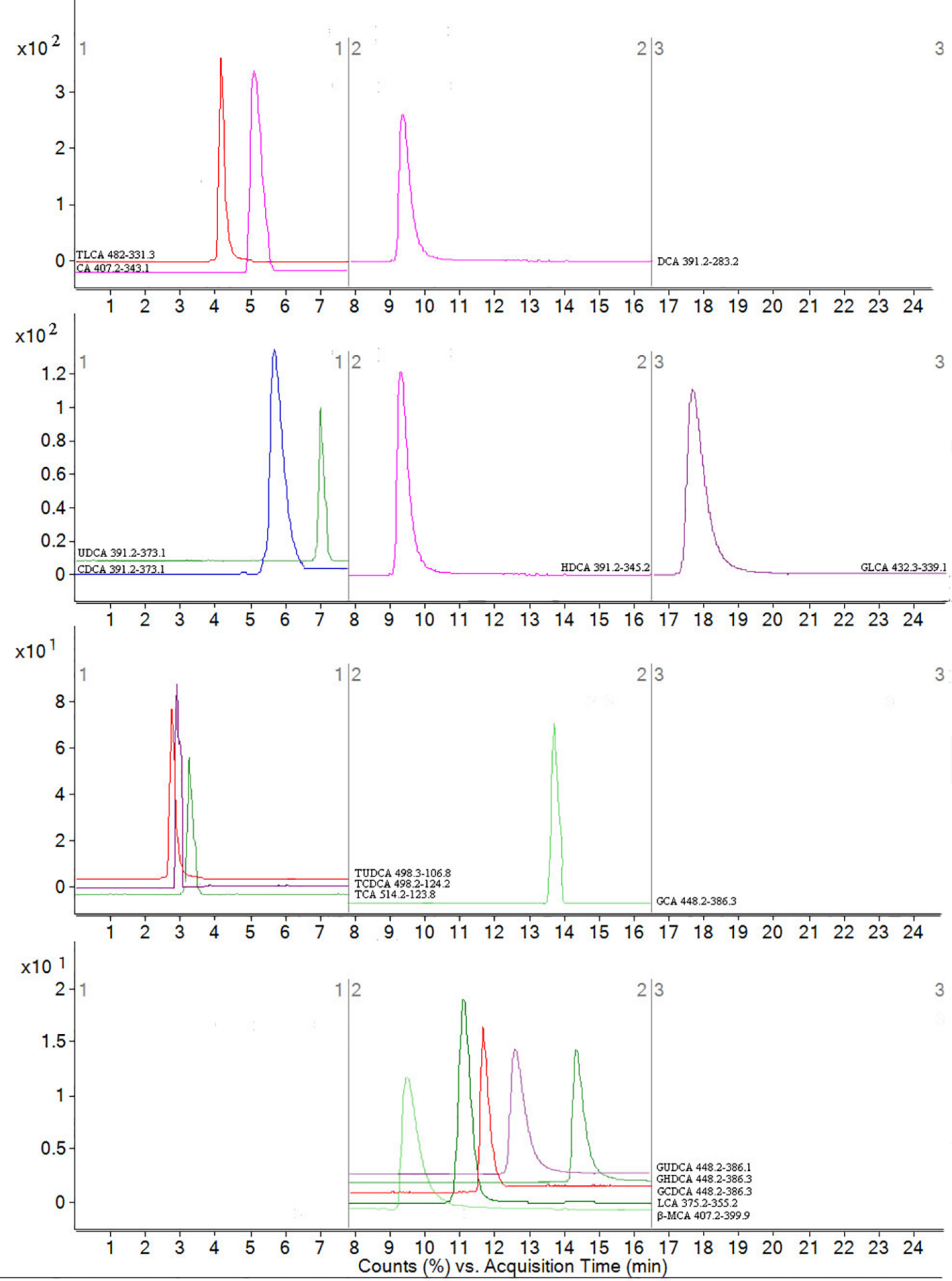
MRM figure for 16 compounds in bile acids. The mass spectrometer was programmed to produce MS/MS spectra in negative ionization modes by ESI source. Scan width for MRM, MRM was performed by using mass transition between specific parent ions into corresponding fragment ions for each analyte. According to the retention time and peak area of each fragment ion, qualitative and quantitative analysis was carried out. The x-coordinate is time, and y-coordinate is the ion strength. 16 bile acids including CA, LCA, DCA, CDCA, β-MCA TCDCA, GCDCA, TCA, GCA, HDCA, GHDCA, TLCA, UDCA, TUDCA, GUDCA and GLCA.

**Figure 5:**
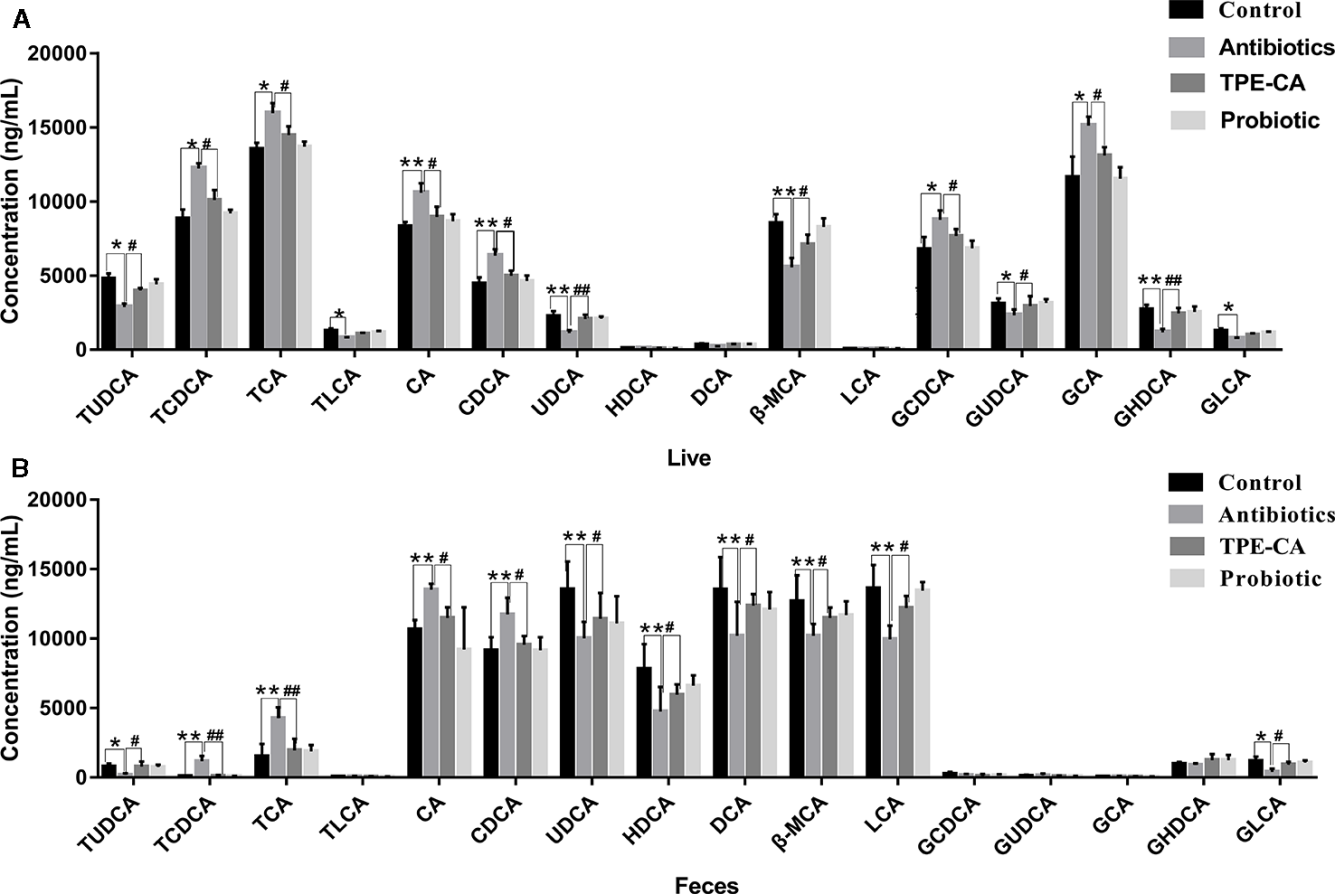
The relative concentrations of 16 bile acid compounds. **(A)** The relative concentrations of 16 compounds in control group, antibiotics group, TPE-CA and probiotic group in liver. **(B)** The relative concentrations of 16 compounds in control group, antibiotics group, TPE-CA and probiotic group in feces. *p < 0.05, **p < 0.01 versus control group. ^#^*p <* 0.05, ^##^*p <* 0.01 versus antibiotics group. Values are represented as means ± S.D. for 10 animals per group. CA, cholic acid; CDCA, chenodeoxycholic acid; β-MCA, beta-muricholic acid; GLCA, glycolithocholic acid; UDCA, ursodeoxycholic acid; HDCA, hyodeoxycholic acid; DCA, deoxycholic acid; TUDCA, tauroursodeoxycholic acid; GCA, glycocholic acid; TLCA, taurolithocholic acid; GCDCA, glycochenodeoxycholic acid; TCA, taurocholic acid; LCA, lithocholic acid; GHDCA, glycohyodeoxycholic acid; GUDCA, glycoursodeoxycholic acid; TCDCA, taurochenodeoxycholic acid.

To assess whether changes in antibiotics induced dysbiosis directly associated with changes in bile acids profiles, 16 differential bile acids, including nine conjugated bile acids and seven free unconjugated bile acids were analyzed using RRLC-QqQ-MS^n^ analysis (Li and Chiang, 2014). Representative MRM chromatograms are shown in **Figure 4**. The detailed changes in individual bile acids are shown in **Figure 5A**, and the specific data are shown in **Table S4**. Comparatively speaking, the proportions of CDCA, CA (*P <* 0.01), TCDCA, GCDCA, TCA and GCA (*P <* 0.05) were increased, while GHDCA, UDCA, β-MAC, (*P <* 0.01), GLCA, TLCA, GUDCA and TUDCA (*P <* 0.05) were decreased in the liver after antibiotics administrations compared to the control group. **Figure 5B** (the specific data shown in **Table S5**) shows enhanced levels of CA, CDCA, TCDCA and TCA (*P <* 0.01), conversely, reduced levels of LCA, HDCA, β-MAC and UDCA (*P <* 0.01), DCA, TUDCA and GLCA (*P <* 0.05) in feces after antibiotics treatment compared to the control group. After treatment with TPE-CA and probiotics, the levels of the bile acids improved, respectively. The TPE-CA group was closest to the normal group. Therefore, TPE-CA restored antibiotics disruption of the enterohepatic circulation of bile acids.

### Regulation of Bile Acid Profiles by TPE-CA Treatment

**Figure 6:**
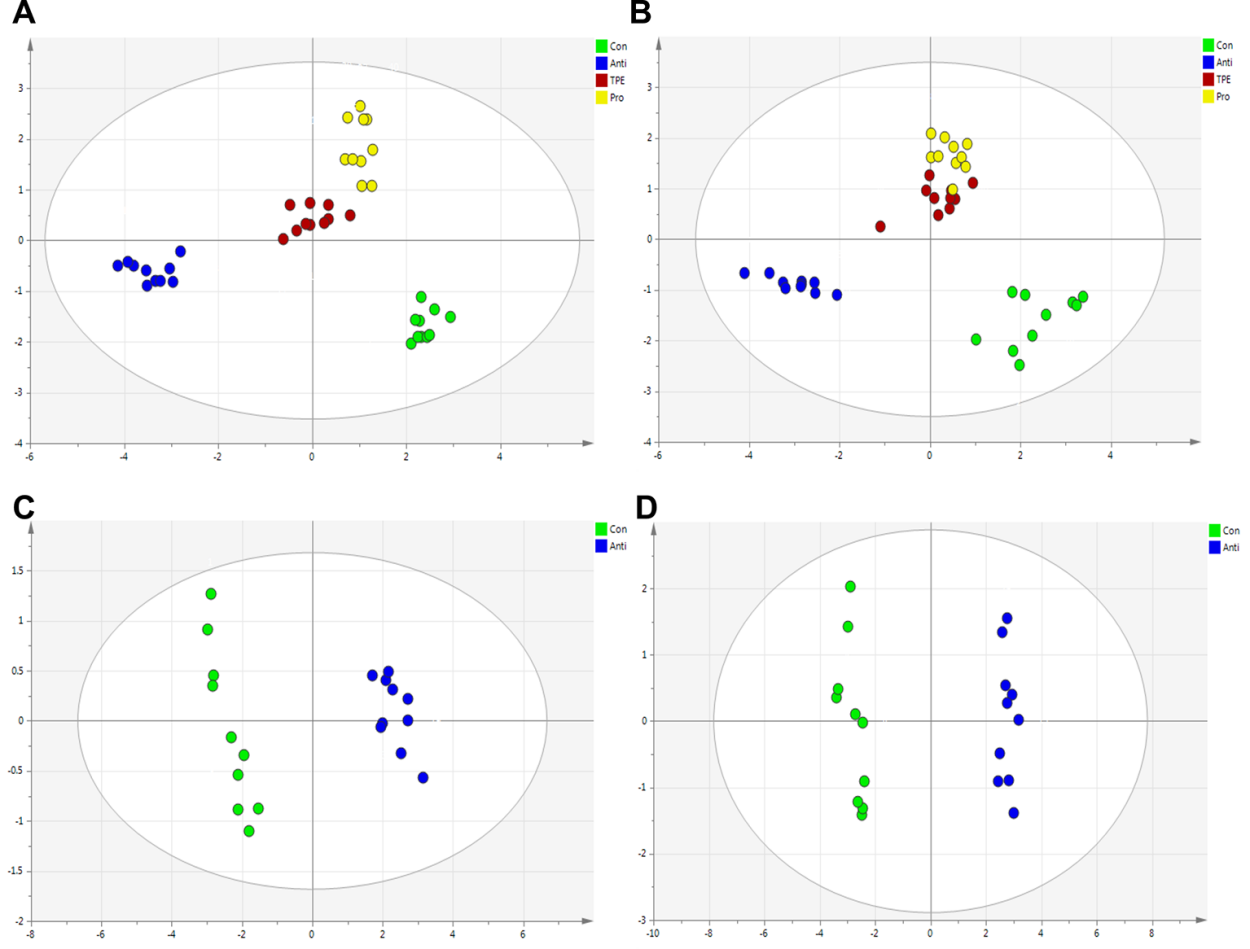
Multivariate statistical analysis of the bile acid compounds. **(A)** PCA score plot of control group, antibiotics group, TPE-CA and probiotic group in liver. **(B)** PCA score plot of control group, antibiotics group, TPE-CA and probiotic group in feces. **(C)** OPLS-DA score plot of control group and antibiotics group in liver. **(D)** OPLS-DA score plot of control group and antibiotics group in feces.

The RRLC-QqQ-MS^n^ date awere input into the SIMCA-P 13.5 software to analyze the differences of bile acid in liver and feces samples of all groups. The PCA scores plot showed the separation between control, antibiotics, TPE-CA and probiotic group were obviously significant (R^2^X = 0.928, Q^2^ = 0.808 and R^2^X = 0.724, Q^2^ = 0.641). (**Figures 6A, B**). The TPE-CA treatment and control groups were close to each other and both groups were separated from the antibiotics group. This analysis showed the effects of treatment with TPE-CA compared to the antibiotics-treated mice. In addition, OPLS-DA results showed a significant separation between the antibiotics-treated group and the control group, revealed that this model was efficient. (R^2^X = 0.829, R^2^Y = 0.72, Q^2^ = 0.671 and R^2^X = 0.792, R^2^Y = 0.987, Q^2^ = 0.977) (**Figures 6C, D**)

### Upregulation of the Liver-Intestinal Axis Related FXR Pathway With TPE-CA Treatment

#### Effect of TPE-CA on FXR/FGF15 Pathway

**Figure 7:**
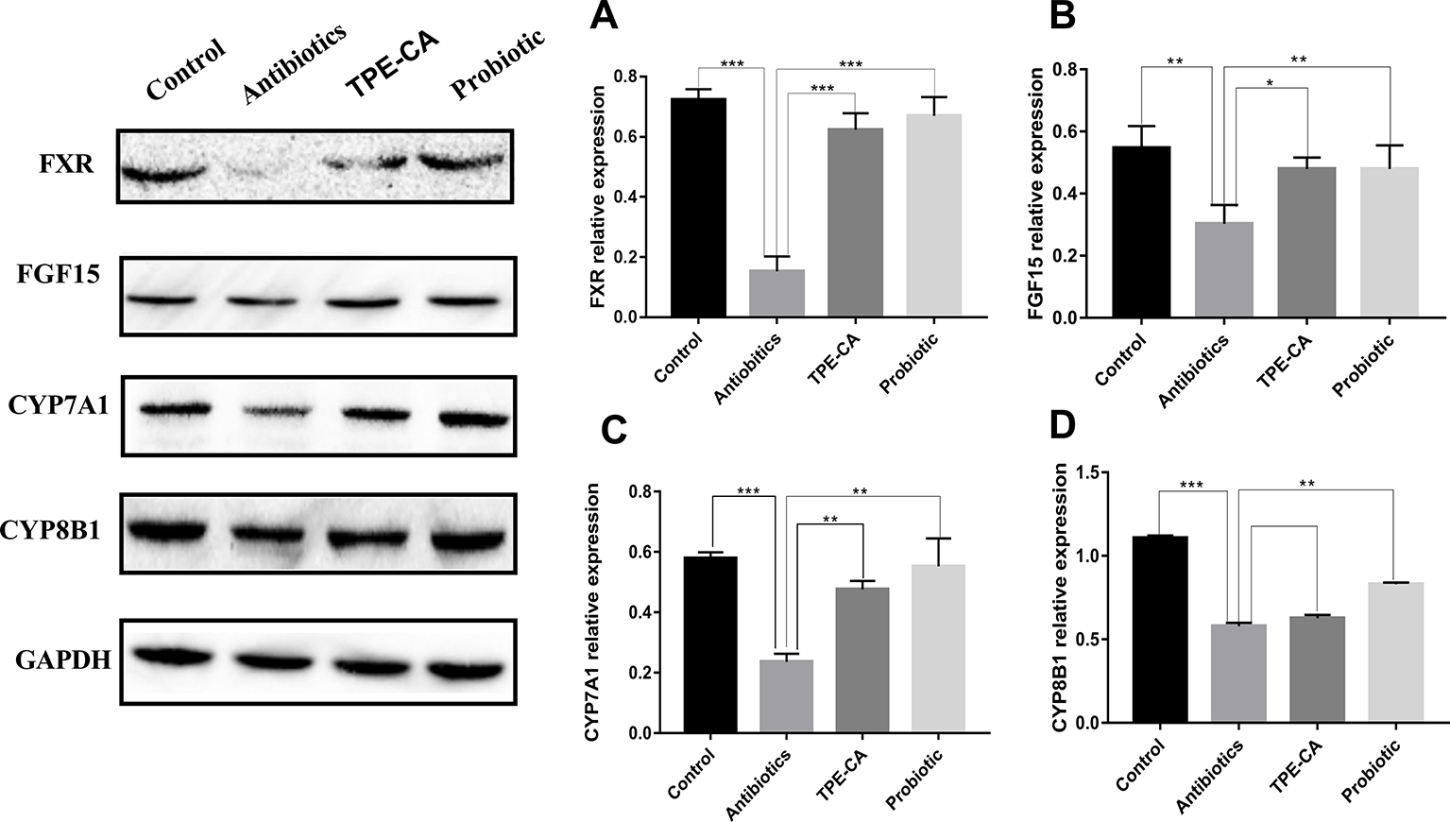
Effect of TPE-CA on intestinal integrity and FXR/FGF15 pathway after antibiotics disturbance. **(A)** The level of FXR in intestinal for four groups. **(B)**The level of FGF15 for four groups. **(C)** The level of CYP7A1 for four groups. **(D)** The level of CYP8B1 for four groups. Expressions are normalized to GAPDH. *p < 0.05, **p < 0.01, ***p < 0.001. Values are represented as means ± S.D for 10 animals per group.

We examined the ileum FXR signaling pathway in animals (Inagaki et al., 2005). FXR expression in the ileum of the antibiotics group was reduced compared to control group (**Figure 7A**). FGF15 reduced, detected in dysbiosis mice compared to controls (**Figure 7B**). Compared to the antibiotics group, the expression of FXR and FGF15 were shown to be significantly up-regulated in ileum treated with the TPE-CA or probiotic. Compared to the antibiotics group, we observed decreased hepatic CYP7A1 protein expression treated with TPE-CE or probiotic (**Figure 7C**). The antibiotics did not affect the protein expression of another enzyme involved in bile acids synthesis, CYP8B1 (**Figure 7D**). This result indicated that TPE-CA attenuates the antibiotics disruption the negative feedback loop on bile acids synthesis by efficiently modulating the ileum FXR-FGF15 pathway.

#### Effect of TPE-CA on FXR-Targeted Protein

**Figure 8:**
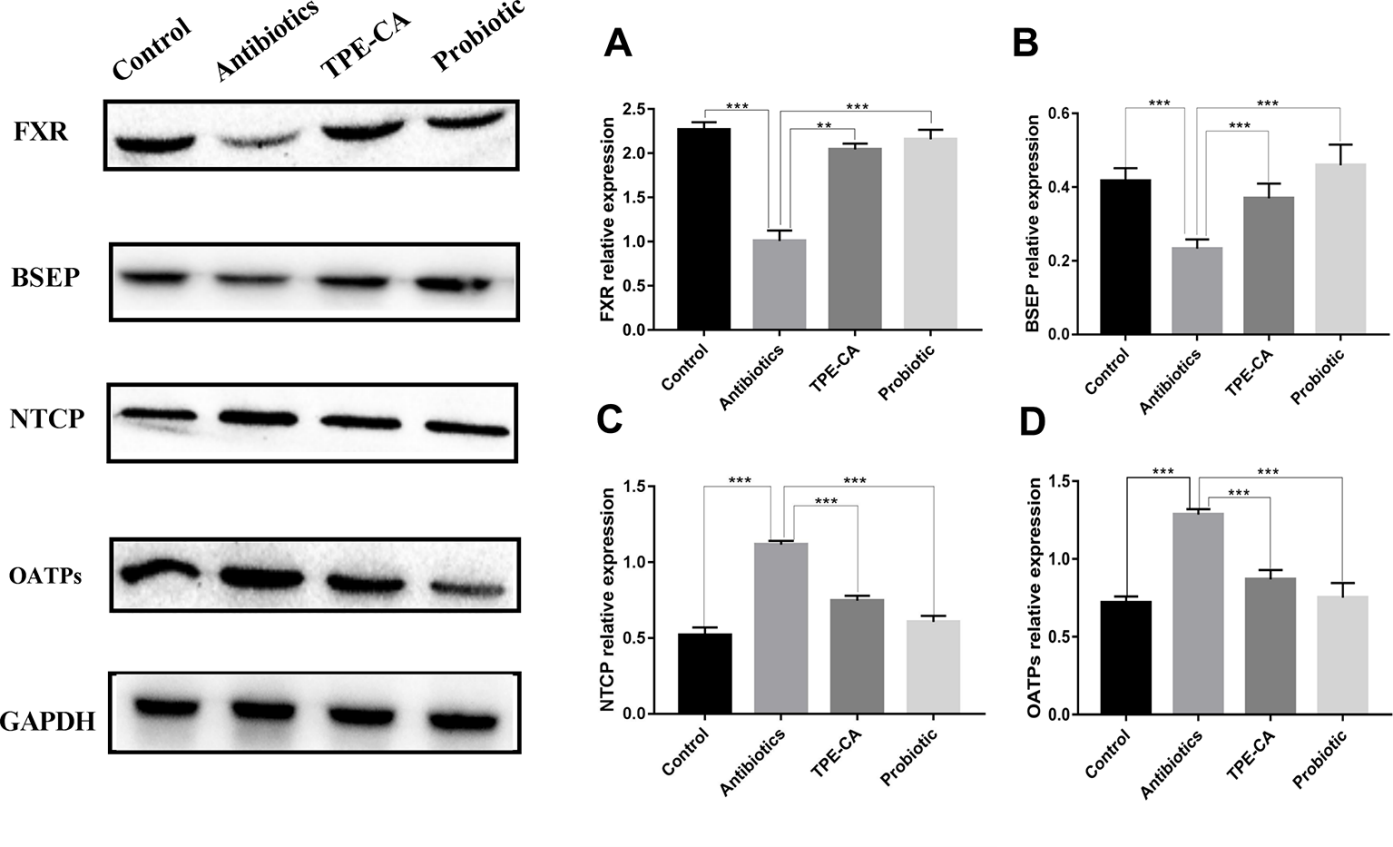
Effect of TPE-CA on FXR-targeted protein. **(A)** The level of FXR in liver for four groups. **(B)** The level of BSEP for four groups. **(C)** The level of NTCP for four groups. **(D)** The level of OATPs for four groups. Expressions are normalized to GAPDH. **p < 0.01, ***p < 0.001. Values are represented as means ± S.D for 10 animals per group.

We examined the protein expression of NTCP, OATPs and BSEP, which are regulated by FXR in the liver. NTCP, OATPs and BSEP are hepatic bile acids transporters. The major functions of NTCP and OATPS are bile acids uptake from the portal circulation to hepatocytes for maintaining enterohepatic circulation, while BSEP mediates the secretion of bile acids, across the canalicular membrane of hepatocytes into gall bladder to provide the osmotic pressure for bile flow (Stieger, 2011; Wolkoff, 2014). As predicted, western blot results showed that the TPE-CA and probiotic groups significantly reversed the downregulation of FXR and BSEP of expression compared with antibiotics groups (**Figures 8A, B**) and the upregulation of NTCP and OATPs (**Figures 8C, D**). Collectively, these results demonstrated that TPE-CA maintained the homeostasis of bile acids *via* regulation of FXR pathway.

## Discussion

**Figure 9:**
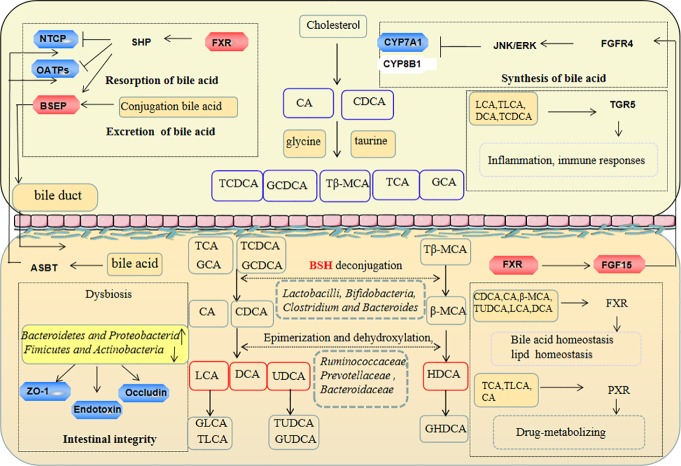
The protection effect of TPE-CA on intestinal microbiota and bile acid metabolism in antibiotics-induced dysbiosis. These include the regulation of barrier integrity, bile acid homeostasis by restoration intestinal microbiota composition and activation liver–gut axis. The red represents the factor being enhanced and the blue represents the weakened factor when treated with TPE-CA.

The gut microbiota plays a key role in the development and preservation of gut barrier integrity and changes in bile acids in liver-gut axis metabolism (Nobel et al., 2015). The present study, established an antibiotics induced gut dysbiosis model, and correlation analyses were used to evaluate the relationship between the microbiota and bile acids. The results revealed that TPE-CA supplementation enhanced microbial diversity, restored the gut microbiota community structure, ameliorated disruption of gut barrier integrity and maintained homeostasis of bile acids metabolism (**Figure 9**).

We analyzed the diversity indices of alpha and beta to investigate microbial diversity, richness, and taxonomic analysis determined differences in intestinal flora between different treatment groups. After antibiotics intervention, the alpha diversity was reduced and beta diversity indices deviated from the control group. Taxonomic analysis revealed that several microbial taxa including *Bacteroidetes* and *Proteobacteria* were found to be increased, and *Fimicutes* and *Actinobacteria* were decreased. Changes in the *Firmicutes/Bacteroides* ratio may be related to body weight loss as a result (Shi et al., 2018a). At the family and genus levels, the relative abundance of *Bacteroides*, *Lachnoclostridium*, *Escherichia–Shigella*, *Enterococcaceae* and *Enterobacteriaceae* increased and *Ruminococcaceae*, *Lactobacillaceae* and *Prevotellaceae* decreased. Patients with intestinal dysbiosis also found a significant decreased in the abundance of *Lachnospiraceae* (Zhao et al., 2017). Literature shows that *Bacteroides* and *Enterobacteriaceae* account for a large proportion in patients with gastroenteritis and irritable bowel syndrome, indicating that there is a significant correlation between (Jalanka-Tuovinen et al., 2014), and the increased relative abundance of *Enterobacteriaceae* and decreased *Lachnospiraceae* were closely related to gut inflammation. The relative abundance of *Bacteroides* and *Proteobacteria* genes such as *Enterococcus* were increased, which belongs to endotoxin-producing bacteria that are harmful to hosts (Villanueva-Millan et al., 2015). The present study demonstrated that, TPE-CA and probiotic supplementation enhanced microbial alpha and beta diversity, and restored the gut microbiota community structure including the reduction of *Proteobacteria*, *Bacteroides* and *Enterococcus* and the enhancement of *Firmicutes*, *Ruminococcaceae, Prevotellaceae* and *Lactobacillaceae*. The level of endotoxin production increased, and ZO-1 and occludin decreased after antibiotics treatment. The reduced expression of proteins is closely related to the permeability of the intestine (Yu et al., 2005). However, TPE-CA treatment reshaped intestinal permeability *via* upregulation of the expression of ZO-1, Occludin proteins and downregulation of serum endotoxin compared with the antibiotics group. Collectively, these results at different taxonomic levels suggest that oral administration of TPE-CA enhance bacterial diversity and richness, and regulate intestinal flora ecology *via* reversion dysbiosis and maintain intestinal integrity.

Bacteria with BSH activity perform bile acids deconjugation. Previous studies demonstrated that functional BSH was present in *Lactobacilli*, *Clostridium*, *Bacteroides* and *Bifidobacteria* (Jones et al., 2008). Lin, 2014 reported a positive correlation between increased BSH activity and weight gain (Lin, 2014). Conjugated bile acids (e.g., TCDCA, GCDCA,TCA, GCA and Tβ-MCA) are transformed to unconjugated bile acids by BSH produced in microbiota, and subjected to chemical modifications of CA to DCA, CDCA to UDCA or LCA and β-MCA to HDCA *via* connection with the intestinal flora (e.g., *Ruminococcaceae*, *Prevotellaceae*, *Bacteroidaceae*) (Ridlon et al., 2006). Antibiotics induced dysbiosis showed increased primary bile acids (e.g., CA and CDCA) and decreased secondary bile acids (e.g., DCA, UDCA, LCA, β-MCA and HDCA) production compared to the control group. TPE-CA and probiotic feeding, remarkably increased BSH and significantly increased the bacterial involved in bile acids metabolism in intestinal bacteria. TPE-CA treatment improved the levels of primary bile acids and secondary bile acids. These results demonstrated that TPE-CA maintained the bile acids homeostasis.

Changes in intestinal flora lead to alterations of the bile acids pool, and the bile acids themselves affect the composition of the intestinal flora. Hydrophobic bile acids, such as CDCA, CA, UDCA, etc., can destroy intestinal membrane and may have an impact on the structure and composition of microbial community (D'aldebert et al., 2009). Studies have shown that feeding mice HDCA significantly increased the abundance of *Firmicutes*, decreased the abundance of *Bacteroides*, and affected the structure of intestinal flora (Joyce et al., 2014). These molecules also activate receptors, such as FXR and TGR5 which regulate several host processes, including energy metabolic processes, glucose metabolic processes, lipid metabolic processes, anti-inflammatory processes(Gonzalez et al., 2015). The most potent ligands for FXR are CDCA, DCA, LCA, CA, GCA and β-MCA. UDCA does not activate FXR; but it inhibits FXR activation. The activation of FXR is involved in glucose and lipid metabolism *via* TUDCA regulation of the cAMP/PKA pathway (Comeglio et al., 2018). TGR5 is activated by multiple bile acids such as LCA, DCA, TLCA and TCDCA. Recent studies have shown that TGR5 ligand decreased the expression of inflammatory cytokines in LPS response (Hogenauer et al., 2014). Conjugated bile acids, including GLCA, GCDCA, GHDCA, GUDCA and TUDCA are amphiphilic molecules that are, present in mixed micelles and have strong lipid emulsifier functions. TLCA, CA and TCA activate PXR, and the main targets of this nuclear receptor are drug-metabolizing enzymes and transporter proteins.

To further confirm the TPE-CA effect on bile acids metabolism, we measured the expression of FXR-FGF15 pathway and FXR-target proteins including BSEP, NTCP and OATPs. FXR induces the expression of small heterodimer partner (SHP) in the liver, which inhibits transcription of CYP7A1 and CYP8B1 induced by liver homolog-1 (LRH-1)-induced gene transcription of CYP7A1 and CYP8B1. NTCP, OATPs and BSEP are regulated *via* a SHP-dependent pathway. Activated FXR in the gut stimulates the intestinal hormone FGF15, and binds to the hepatocyte surface fibroblast growth factor receptor 4 (FGFR4) to activate extracellular signal-regulated kinase (ERK) in humans and Jun N-terminal kinase (JNK) in mice mediates the suppression of CYP7A1 and CYP8B1 gene expression (Kong et al., 2018). TPE-CA treatment showed increased the expression of FXR and FGF15 and decreased the expression of CYP7A1 compared to the antibiotics group in the present study. However, the expression of CYP8B1 was not significantly different. These results indicated modulation of the liver-gut axis by FXR-FGF15-CYP7A1 pathway after TPE-CA treatment. Due to the activated FXR-FGF15 pathway, conjugated bile acids (TCDCA, GCDCA, TCA and GCA) and unconjugated bile acids (CA and CDCA) were decreased in the liver. Therefore, TPE-CA mediates the synthesis of bile acids through the negative feedback regulatory mechanism by FXR pathway. Bile acids, mainly conjugated bile acids (GUDCA, GLCA, TLCA, GHDCA and TUDCA) are taken up by NTCP and OATPs at the basolateral membrane of hepatocytes. The monovalent bile acids are excreted into the bile canaliculus *via* BSEP. After antibiotics treatment, the protein expression levels of NTCP and OATPs were increased, and the expression level of BSEP was decreased. These results suggest that alterations in the intestinal flora markedly influence the protein expression of these molecules. However, the results were reversed in the TPE-CA and probiotic groups. NTCP, OATPs and BSEP were regulated through FXR pathway *via* the SHP-dependent. TPE-CA mediates the expression of bile acid transporter by regulating the FXR pathway, inhibits the uptake of bile acid, promotes the secretion of bile acid, and maintains the stable state of bile acid. Overall, TPE-CA regulated bile acids enterohepatic circulation and maintained bile acids homeostasis *via* FXR signaling modulation the liver-gut axis.

## Conclusion

In conclusion, he results of the present study indicated that TPE-CA effectively improved the intestinal microbiota composition using a metagenomics analysis and exhibited a protective effect on barrier integrity *via* increased the expression of ZO-1, occludin and decreased endotoxin production. TPE-CA regulated bile acids entry into the enterohepatic circulation and maintained bile acids homeostasis *via* FXR signaling modulation the liver–gut axis. Natural substances such as TPE-CA may alternative and complementary medicine therapy for dysbiosis in the future. The molecular mechanisms and the exact treatment doses of TPE-CA must be elucidated to achieve the best benefit profile.

## Data Availability Statement

The raw data supporting the conclusions of this article will be made available by the authors, without undue reservation, to any qualified researcher.

## Ethics Statement

Specific pathogen-free (SPF) grade laboratory was carried out in accordance with the Guide for the Care and Use of Laboratory Animals. The rodent license of the laboratory (NO. SYXK 11-00-0039) was issued by the National Science and Technology Ministry of China. All animals' protocols were approved by the Animal Center of the Institute of Basic Theory, China Academy of Chinese Medical Sciences.

## Author Contributions

LL contributed to the acquisition and analyses of data for the work, and to drafting of the manuscript. ZL contributed to the conception and design of the work. Revising the work critically for important intellectual content, HL contributed to the organization of the contents of the manuscript, and participated in the discussion on views in the paper. ZC, WL, ZS and XL contributed to the acquisition and analyses of data for the work, and participated in the discussion on views in the paper. AL, CL and YL contributed to the conception and design of the work and revised the work critically for important intellectual content. All authors read and approved the final manuscript.

## Conflict of Interest

The authors declare that the research was conducted in the absence of any commercial or financial relationships that could be construed as a potential conflict of interest.

## Abbreviations

TPE-CA, Total phenolic extracts of *Citrus aurantium* L.; ZO-1, Zonula occludens 1; FXR, Farnesoid X receptor; FGF15, Fibroblast growth factor 15; CYP7A1, Cholesterol 7α-hydroxylation; CYP8B1, Sterol 12α-hydroxylase; BSEP, Bile salt export pump, NTCP, Na+-taurocholate cotransporting polypeptide; OATPs, Organic anion transporters; ASBT, Apical sodium dependent bile acid transporter; BSH, Bile salt hydrolase; TGR5, The G protein-coupled bile acid receptor 1; JNK, Jun N-terminal kinase; LRH-1, Liver homolog-1; SHP, Small heterodimer partner; ELISA, Enzyme-linked immunosorbent assay.
