# Supplementary material for: Naturally Occurring TPE-CA Maintains Gut Microbiota and Bile Acids Homeostasis via FXR Signaling Modulation of the Liver–Gut Axis
Source: Front Pharmacol. 2020 Feb 6;11:12. doi: 10.3389/fphar.2020.00012 (PMC7015895; doi:10.3389/fphar.2020.00012)
Supplement: Supplementary file 1 [file DataSheet_1.pdf]

Supplementary Material

Naturally occurred TPE-CA maintain gut microbiota and bile acids homeostasis through FXR signaling modulated liver - gut axis

Contents:

Table S1. The specific contents of 32 phenolic compounds
Table S2. The information for MRM parameters.
Table S3. Calibration curves, correlation coefficients, linear ranges and LOQs of 16 compounds
Table S4. The measurement results of 16 bile acid compounds in liver.
Table S5. The measurement results of 16 bile acid compounds in feces.
Table S6 The raw tags and clean tags in each sample.
Table S7 The sequences distribution statistics in differences length.
Table S8 The OTUs statistics in each sample.
Table S9. The Chao1 and Shannon of alpha diversity index in each sample.
Figure S1: The HPLC-based chemprofile of total phenolic extracts of *Citrus aurantium* L
Figure S2. The relative abundance of bacterial phylum in each samples.
Figure S3. The relative abundance of bacterial class in each samples.
Figure S4. The relative abundance of bacterial order in each samples.
Figure S5. The relative abundance of bacterial family in each samples.
Figure S6. The relative abundance of bacterial genus in each samples.
Figure S7. The genus_phylogeny for each groups.
Figure S8. The co-occurrence network for OTUs.

Table S1. The specific contents of 32 phenolic compounds.

NO	Name	Content (ng/ml)
1	Acacetin	770.42
2	Apigenin	1490.18
3	Apigenin-7-O-Glucoside	19076.58
4	Auraptene	1595.34
5	Bergapten	1653.63
6	5-Demethylnobiletin	113856.18
7	Diosmetin	5558.46
8	Diosmetin-7-O-Glucoside	34483.38
9	Diosmin	629279.22
10	Eriocitrin	2166024.54
11	Eriodictyol	4218.94
12	Hesperetin	340966.86
13	Hesperidin	20603969.13
14	Imperatorin	874.38
15	Isopimpinellin	3898.14
16	Isosakuranetin	792.48
17	Kaempferitrin	9062.52
18	Limonin	97171.56
19	Luteolin	2591288.19
20	Naringenin	261388.26
21	Naringin	61045773.93
22	Narirutin	17752304.46
23	Neohesperidin	97244453.52
24	Nobiletin	2291214.32
25	Nomilin	50669.22
26	Poncirin	520566.96

27	Rhoifolin	2631472.98
28	Rutin	852041.22
29	Scoparone	4124.04
30	Sinensetin	256528.62
31	Tangeretin	3802648.92
32	Xanthotoxol	15009.26

Table S2. The information for MRM parameters.

No	Compound	tR(min)	[M + H]+ (m/z)	MRM Transitions (Precursor↑ → Product)	Fragmentor (V)	Collision Energy (eV)
			Time segments: 0~7.8 min			
01	tauroursodeoxycholic acid	2.882	498.3	498.3→106.8	140	73
02	taurochenodeoxycholic acid	2.901	498.2	498.2→124.2	135	55
03	taurocholic acid	3.039	514.2	514.2→123.8	135	60
04	taurolithocholic acid	4.145	401.3	401.3→331.3	130	20
05	cholic acid	5.122	407.2	407.2→343.1	130	40
06	chenodeoxycholic acid	5.706	391.2	391.2→373.1	175	35
07	ursodeoxycholic acid	6.888	391.1	391.1→373.1	175	20
			Time segments: 7.8~16.5min			
08	hyodeoxycholic acid	9.280	391.2	391.2→345.2	135	30
09	deoxycholic acid	9.306	391.2	391.2→283.2	150	45
10	beta-muricholic acid	9.625	407.2	407.2→399.9	130	40
11	lithocholic acid	11.092	375.2	375.2→375.2	160	50
12	glycochenodeoxycholic acid	11.625	448.2	448.2→386.3	125	40

13	glycoursodeoxycholic acid	12.661	448.2	448.2→386.1	155	35
14	glycocholic acid	13.669	463.1	464.2→402.2	130	40
15	glycohyodeoxycholic acid	14.322	448.2	448.2→386.3	115	10
			Time segments: 16.5~25 min			
16	glycolithocholic acid	17.593	432.3	432.3→432.3	155	20

Table S3. Standard curves, R^2 ,linear range, LOD and LOQ values for 16 compounds.

No	Compound	Standard curve	R^2	Linear range,（ng/mL）	LLOQ（ng/mL）
01	tauroursodeoxycholic acid	y=54.1x-9.183	0.9973	50.00-10200.00	5.34
02	taurochenodeoxycholic acid	y=35.75x-6.391	0.9966	15.00.00-2850.00	7.46
03	taurocholic acid	y=7.2571x+1.3857	0.9952	100.00-30200.00	10.52
04	taurolithocholic acid	y=7.665x-6.512	0.9987	30.00-5500.00	7.19
05	cholic acid	y=2.398x+0.231	0.9952	200.00-20480.00	24.31
06	chenodeoxycholic acid	y=139.6x-75.46	0.9919	125.00-25000.00	83.86
07	ursodeoxycholic acid	y=7.38x-2.99	0.9954	12.50-30000.00	5.61
08	hyodeoxycholic acid	y=17.58x-3.52	0.9948	15.00-25000.00	5.69
09	deoxycholic acid	y=23.89x-15.81	0.9948	50.00-30000.00	34.56
10	beta-muricholic acid	y=7.04x-4.522	0.9963	500.00-20500.00	45.32
11	lithocholic acid	y=9.958x-35.893	0.9977	12.00-25000.00	6.34

12	glycochenodeoxycholic acid	y=25.3x-14.4	0.9923	50.00-2500.00	19.47
13	glycoursodeoxycholic acid	y=20.3x+17.2	0.9963	50.00-18000.00	20.87
14	glycocholic acid	y=15.85x+15.21	0.9963	20.00-35000.00	12.09
15	glycohyodeoxycholic acid	y=3.806x-3.026	0.9926	50.00-15000.00	13.24
16	glycolithocholic acid	y=124.73x-69.31	0.9997	50.00-15000.00	29.37

Table S4. The measurement results of 16 bile acid compounds in liver.

No	Compound	Control (ng/ml)	Antibiotic (ng/ml)	TPE-CA(ng/ml)	Probiotic(ng/ml)
01	tauroursodeoxycholic acid	4782.12±896.4	2328.78±346.92	3988.36±547.36	4418.26±327.19
02	taurochenodeoxycholic acid	8851.25±1023.78	12234.26±847.63	10105.36±1023.47	9196.36±874.61
03	taurocholic acid	13537.72±1348.47	15965.21±1478.62	14482.26±1237.81	13707.64±1023.67
04	taurolithocholic acid	1286.57±95.74	783.56±84.63	1075.69±56.39	1182.69±64.17
05	cholic acid	83330.75±1025.49	10588.74±964.53	8971.54±759.61	8673.75±547.62
06	chenodeoxycholic acid	4456.78±579.64	6346.75±597.42	4993.48±504.36	4637.26±349.52
07	ursodeoxycholic acid	2270.26±345.87	1134.26±102.36	2086.49±89.7	2088.12±105.61
08	hyodeoxycholic acid	109.76±15.62	97.20±6.38	102.36±12.34	105.39±9.68
09	deoxycholic acid	349.24±26.57	208.94±18.36	345.97±21.94	353.59±14.26
10	beta-muricholic acid	8549.26±1347.25	5548.26±749.64	7109.69±648.17	8283.54±947.54
11	lithocholic acid	75.64±7.84	43.29±3.67	67.59±5.39	75.49±13.47

12	glycochenodeoxycholic acid	6785.49±874.36	8763.51±701.64	7672.39±601.57	6852.21±701.49
13	glycoursodeoxycholic acid	3109.52±347.58	2331.96±187.58	2953.92±213.47	3156.23±472.59
14	glycocholic acid	11644.57±1456.81	15119.57±1367.59	12114.29±1269.54	11540.32±1056.94
15	glycohyodeoxycholic acid	2725.67±567.25	1179.29±254.67	2451.62±159.68	2548.51±347.26
16	glycolithocholic acid	1291.25±12.38	749.68±15.67	1014.69±1826	1164.89±23.67

Table S5. The measurement results of 16 bile acid compounds in feces.

No	Compound	Control (ng/ml)	Antibiotic (ng/ml)	TPE-CA(ng/ml)	Probiotic(ng/ml)
01	tauroursodeoxycholic acid	794.26±57.26	193.57±23.57	750.24±102.34	752.31±97.49
02	taurochenodeoxycholic acid	87.26±5.61	570.34±54.68	129.54±16.54	90.52±24.15
03	taurocholic acid	1520.39±84.69	4276.59±564.62	1952.37±364.95	1899.62±315.26
04	taurolithocholic acid	50.74±10.37	69.71±18.62	54.39±9.64	57.59±14.25
05	cholic acid	11650.29±784.59	8927.52±1026.53	10678.26±964.26	11201..68±1026.54
06	chenodeoxycholic acid	9158.71±1071.27	5273.48±479.62	8563.59±1314.21	9158.64±697.16
07	ursodeoxycholic acid	10021.36±1145.67	13538.21±2357.69	11419.26±947.54	11091.52±1025.49
08	hyodeoxycholic acid	4759.61±947.68	7831.47±247.42	5729.47±649.51	5205.69±399.42
09	deoxycholic acid	10211.24±847.26	13518.26±1479.36	10873.71±743.19	9934.57±846.56
10	beta-muricholic acid	12686.46±1204.36	10952.47±947.26	11419.28±1257.36	11690.53±1159.26
11	lithocholic acid	13617.59±1056.72	9950.64±679.24	12610.37±847.94	13453.69±1024.62

12	glycochenodeoxycholic acid	257.42±12.36	175.47±34.57	171.36±45.61	161.54±34.16
13	glycoursodeoxycholic acid	70.64±9.47	163.53±34.51	89.25±15.69	83.47±10.38
14	glycocholic acid	50.54±6.78	69.71±6.49	54.12±7.16	54.67±9.59
15	glycohyodeoxycholic acid	853.71±64.52	2276.57±356.51	1250.57±179.54	961.58±102.79
16	glycolithocholic acid	1185.57±132.41	404.52±26.51	914.52±34.59	1081.52±41.26

Table S6. The raw tags and clean tags in each sample.

NO	Sample	Raw tags	Clean tags
01	control 1	95654	94207
02	control 2	48141	47246
03	control 3	66487	65207
04	control 4	79365	77633
05	control 5	59503	58508
06	control 6	42010	41349
07	control 7	60365	59530
08	control 8	76919	75734
09	control 9	75575	73048
10	control 10	77178	76180
11	antibiotics 1	31712	31218
12	antibiotics 2	24293	23904
13	antibiotics3	23748	23391
14	antibiotics 4	39213	37555
15	antibiotics 5	45316	43081
16	antibiotics 6	93752	86231
17	antibiotics 7	62060	59366
18	antibiotics 8	27282	26774
19	antibiotics9	32744	32263
20	antibiotics 10	65104	62871
21	TPE-CA 1	138391	135891
22	TPE-CA 2	78091	76191
23	TPE-CA 3	120162	116821
24	TPE-CA 4	77249	74327
25	TPE-CA 5	124106	121697

26	TPE-CA 6	83174	80413
27	TPE-CA 7	131219	127120
28	TPE-CA 8	124181	121288
29	TPE-CA 9	95602	93670
30	TPE-CA 10	57956	56399
31	probiotic 1	77121	73470
32	probiotic 2	118121	111438
33	probiotic 3	198421	195495
34	probiotic 4	68828	67949
35	probiotic 5	62197	61493
36	probiotic 6	251817	245856
37	probiotic 7	237388	233363
38	probiotic 8	116590	114175
39	probiotic 9	181164	177208
40	probiotic 10	143089	140946

Table S7. The sequences distribution statistics in differences length.

Length (bp)	Sequences
0-200	0
200-260	0
260-320	42
320-360	68
360-380	26
380-400	1088
400-420	2077902
420-440	1441362
440-460	15
460-480	1
480-500	0
500-520	2
520-540	0
540-560	0
560-600	0

Table S8. The OTUs statistics in each sample.

NO	Sample	Final tags	Final OTUs
01	control 1	20460	501

02	control 2	20460	512
03	control 3	20460	555
04	control 4	20460	544
05	control 5	20460	446
06	control 6	20460	526
07	control 7	20460	507
08	control 8	20460	524
09	control 9	20460	491
10	control 10	20460	475
11	antibiotics 1	20460	37
12	antibiotics 2	20460	39
13	antibiotics3	20460	44
14	antibiotics 4	20460	36
15	antibiotics 5	20460	35
16	antibiotics 6	20460	28
17	antibiotics 7	20460	63
18	antibiotics 8	20460	32
19	antibiotics9	20460	46
20	antibiotics 10	20460	65
21	TPE-CA 1	20460	355
22	TPE-CA 2	20460	432
23	TPE-CA 3	20460	464
24	TPE-CA 4	20460	353
25	TPE-CA 5	20460	404
26	TPE-CA 6	20460	383
27	TPE-CA 7	20460	269
28	TPE-CA 8	20460	417
29	TPE-CA 9	20460	261
30	TPE-CA 10	20460	475
31	probiotic 1	20460	429
32	probiotic 2	20460	451
33	probiotic 3	20460	497
34	probiotic 4	20460	550
35	probiotic 5	20460	465
36	probiotic 6	20460	560
37	probiotic 7	20460	534
38	probiotic 8	20460	520
39	probiotic 9	20460	487
40	probiotic 10	20460	482

Table S9. The Chao1 and Shannon of alpha diversity index in each sample.

NO	Sample	Chao 1	Shannon
01	control 1	593.12	6.87
02	control 2	585.59	6.55
03	control 3	682.85	6.82
04	control 4	622.44	6.95
05	control 5	520.69	6.68
06	control 6	610.26	6.78
07	control 7	587.72	6.75
08	control 8	612.80	6.98
09	control 9	591.94	6.49
10	control 10	572.83	6.58
11	antibiotics 1	38.69	3.18
12	antibiotics 2	47.95	3.57
13	antibiotics3	48.69	3.59
14	antibiotics 4	45.83	2.59
15	antibiotics 5	35.65	3.60
16	antibiotics 6	31.67	3.17
17	antibiotics 7	78.55	3.08
18	antibiotics 8	45.50	3.25
19	antibiotics9	84.38	2.74
20	antibiotics 10	72.52	3.91
21	TPE-CA 1	458.05	6.64
22	TPE-CA 2	496.30	6.89
23	TPE-CA 3	526.19	6.48
24	TPE-CA 4	396.21	6.57
25	TPE-CA 5	471.19	6.65
26	TPE-CA 6	431.35	6.48
27	TPE-CA 7	334.14	5.60
28	TPE-CA 8	475.07	6.61
29	TPE-CA 9	293.09	5.57
30	TPE-CA 10	531.14	6.92
31	probiotic 1	488.26	6.38
32	probiotic 2	518.45	6.47
33	probiotic 3	571.52	6.85
34	probiotic 4	640.15	7.08
35	probiotic 5	519.84	6.77

36	probiotic 6	639.56	7.07
37	probiotic 7	606.17	7.14
38	probiotic 8	609.58	6.54
39	probiotic 9	557.20	6.96
40	probiotic 10	568.22	6.71

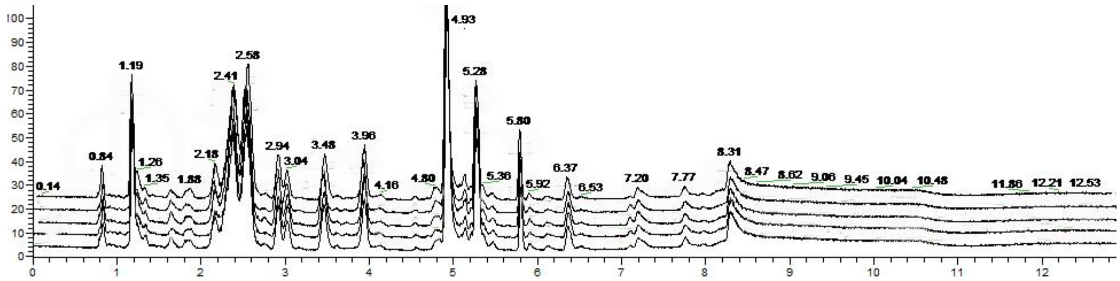

Figure S1. The HPLC-based chemprofile of Citrus *aurantium* L

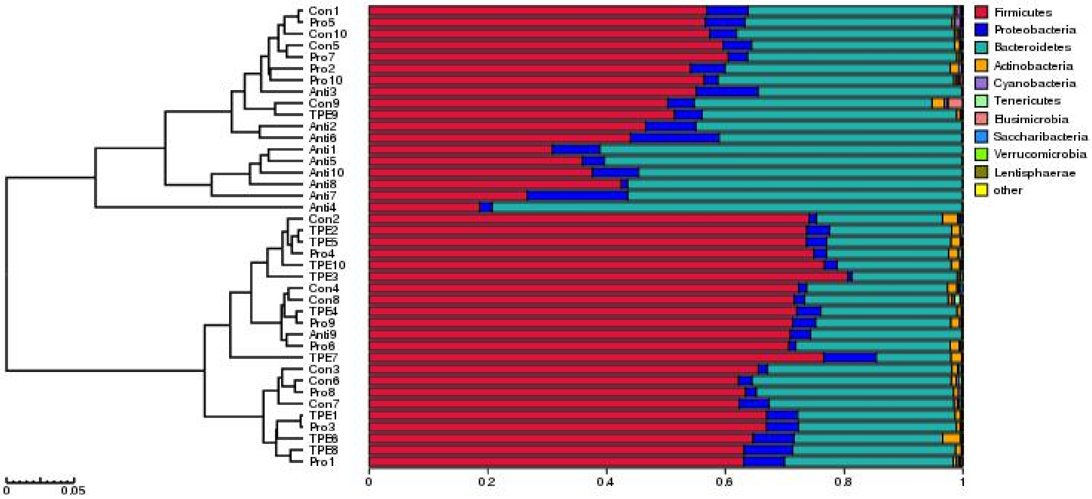

Figure S2. The relative abundance of bacterial phylum in each samples.

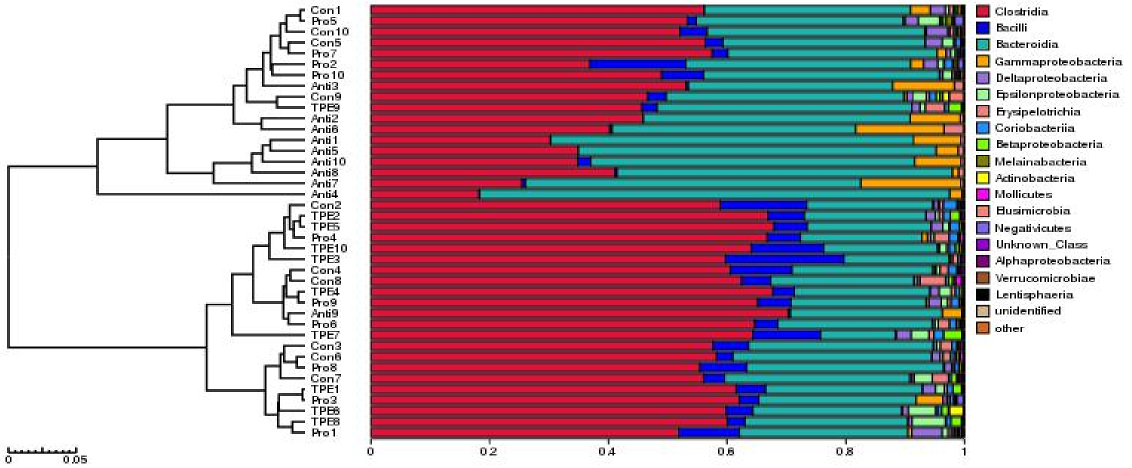

Figure S3. The relative abundance of bacterial class in each samples.

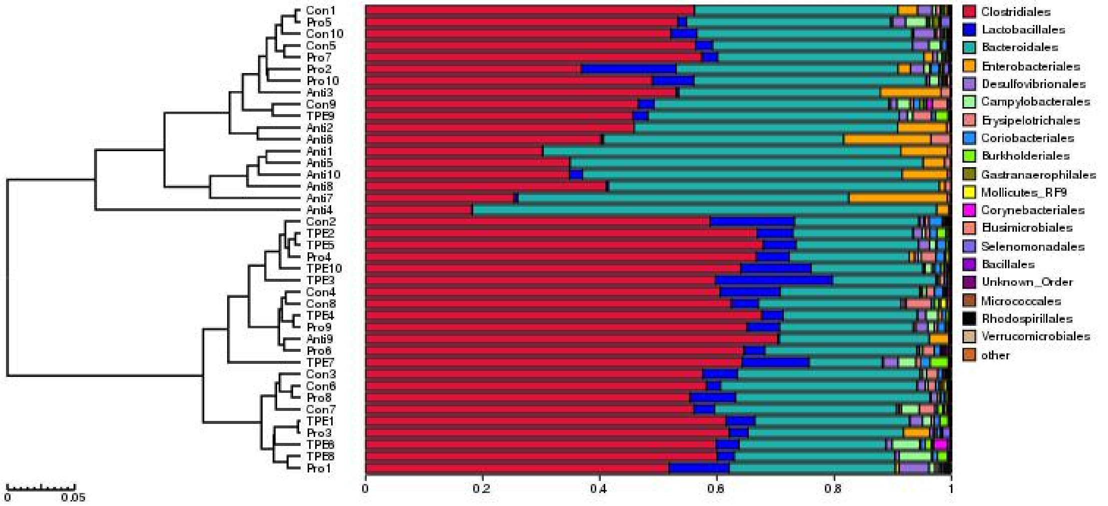

Figure S4. The relative abundance of bacterial order in each samples.

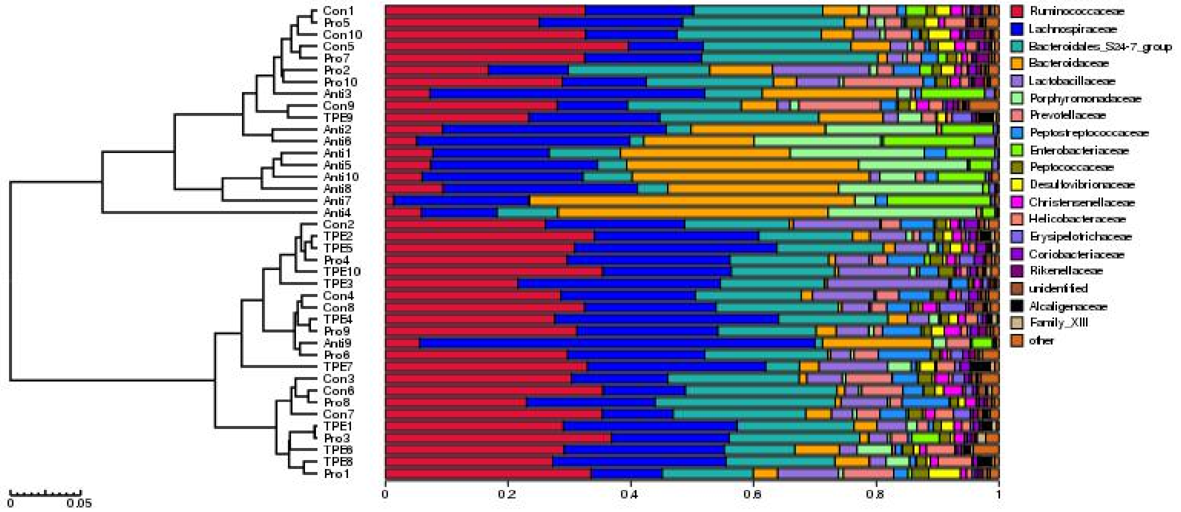

Figure S5. The relative abundance of bacterial family in each samples.

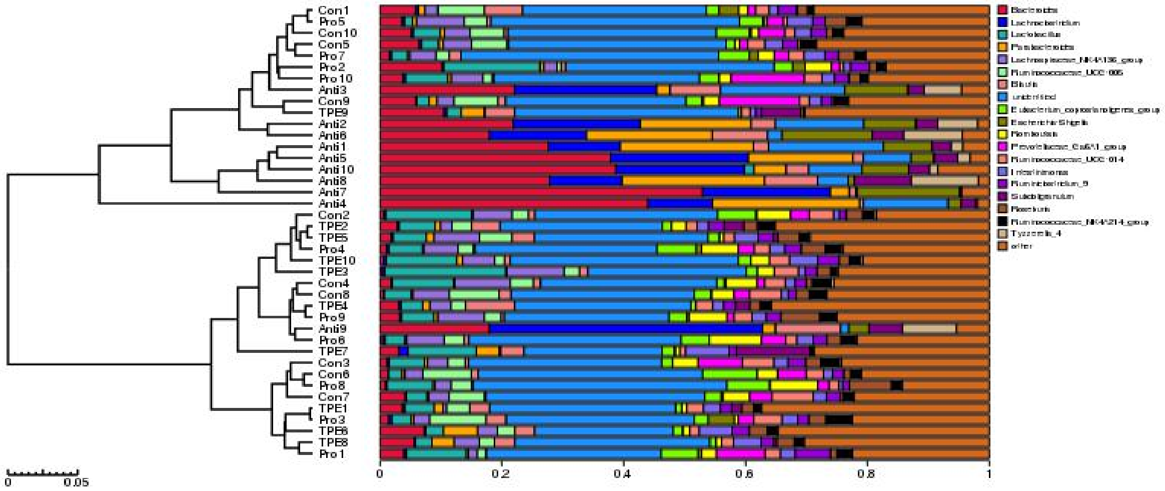

Figure S6. The relative abundance of bacterial genus in each samples.

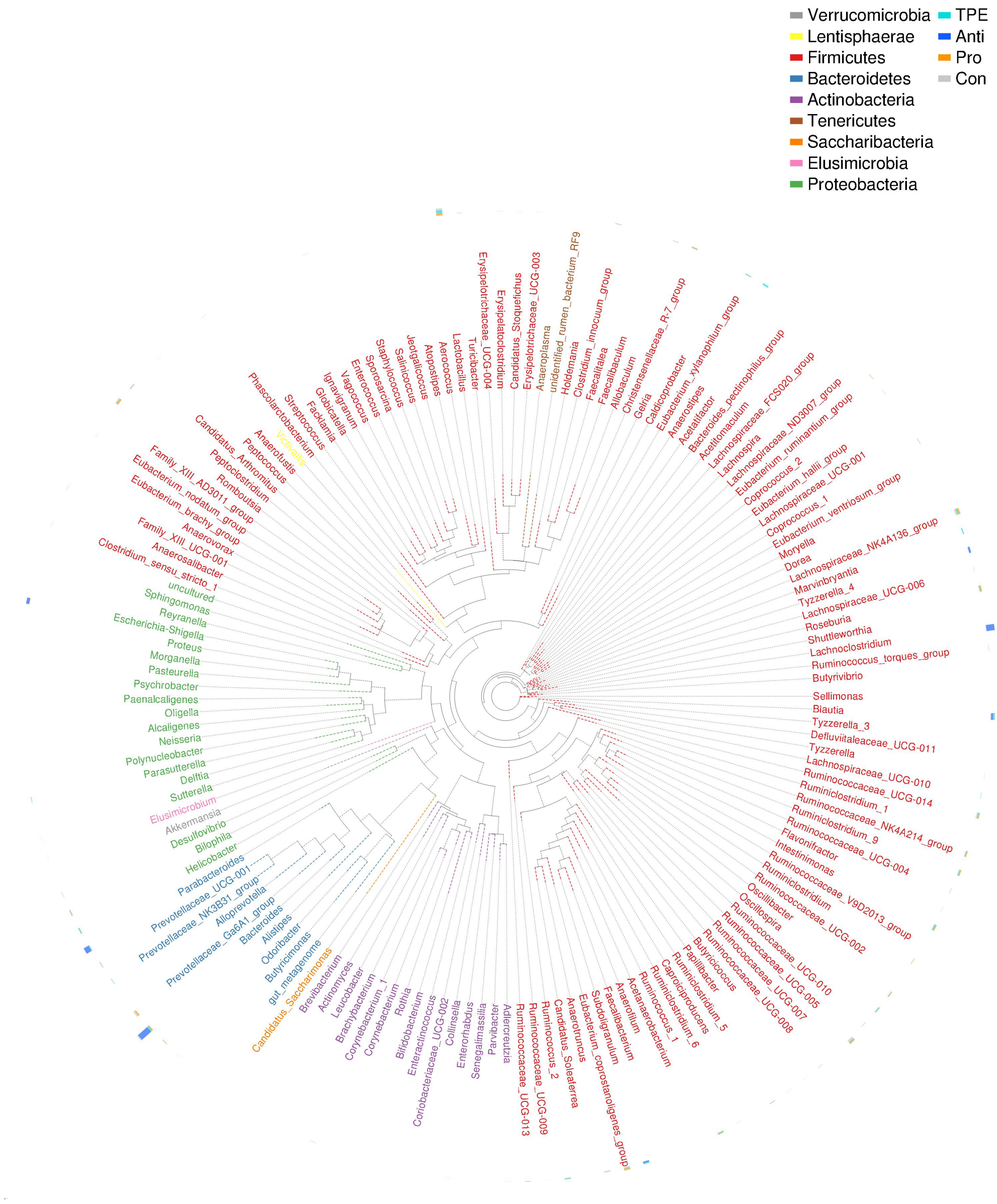

Figure S7. The genus_phylogeny for each groups.

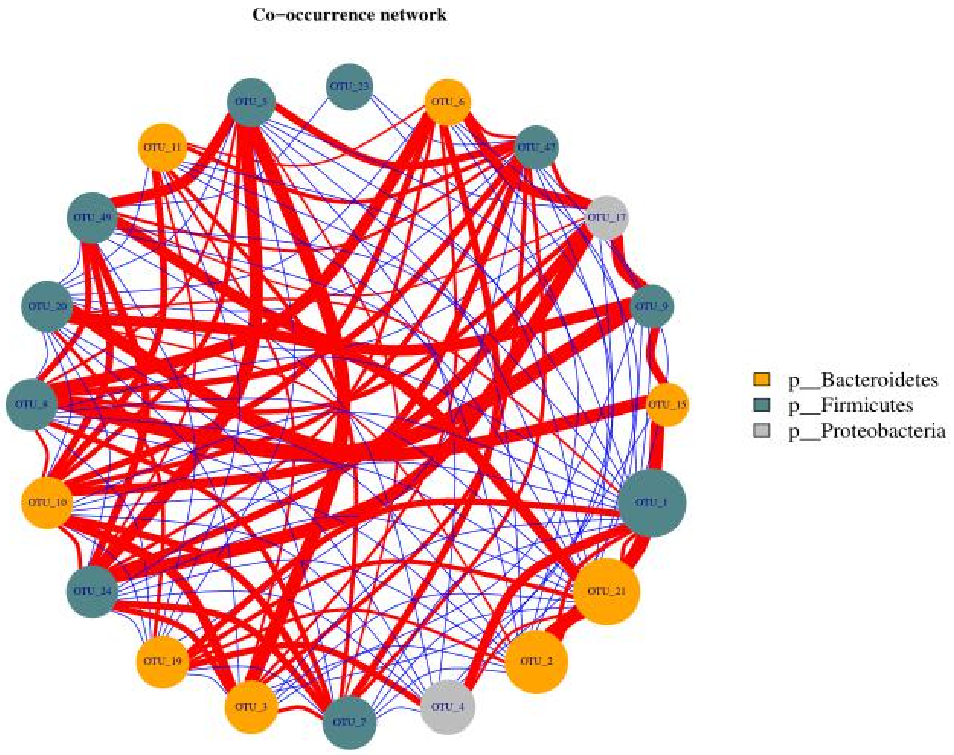

Figure S8. The co-occurrence network for OTUs
